# Meta-analysis of genome-wide association studies discovers multiple loci for chronic lymphocytic leukemia

**DOI:** 10.1038/ncomms10933

**Published:** 2016-03-09

**Authors:** Sonja I. Berndt, Nicola J. Camp, Christine F. Skibola, Joseph Vijai, Zhaoming Wang, Jian Gu, Alexandra Nieters, Rachel S. Kelly, Karin E. Smedby, Alain Monnereau, Wendy Cozen, Angela Cox, Sophia S. Wang, Qing Lan, Lauren R. Teras, Moara Machado, Meredith Yeager, Angela R. Brooks-Wilson, Patricia Hartge, Mark P. Purdue, Brenda M. Birmann, Claire M. Vajdic, Pierluigi Cocco, Yawei Zhang, Graham G. Giles, Anne Zeleniuch-Jacquotte, Charles Lawrence, Rebecca Montalvan, Laurie Burdett, Amy Hutchinson, Yuanqing Ye, Timothy G. Call, Tait D. Shanafelt, Anne J. Novak, Neil E. Kay, Mark Liebow, Julie M. Cunningham, Cristine Allmer, Henrik Hjalgrim, Hans-Olov Adami, Mads Melbye, Bengt Glimelius, Ellen T. Chang, Martha Glenn, Karen Curtin, Lisa A. Cannon-Albright, W Ryan Diver, Brian K. Link, George J. Weiner, Lucia Conde, Paige M. Bracci, Jacques Riby, Donna K. Arnett, Degui Zhi, Justin M. Leach, Elizabeth A. Holly, Rebecca D. Jackson, Lesley F. Tinker, Yolanda Benavente, Núria Sala, Delphine Casabonne, Nikolaus Becker, Paolo Boffetta, Paul Brennan, Lenka Foretova, Marc Maynadie, James McKay, Anthony Staines, Kari G. Chaffee, Sara J. Achenbach, Celine M. Vachon, Lynn R. Goldin, Sara S. Strom, Jose F. Leis, J. Brice Weinberg, Neil E. Caporaso, Aaron D. Norman, Anneclaire J. De Roos, Lindsay M. Morton, Richard K. Severson, Elio Riboli, Paolo Vineis, Rudolph Kaaks, Giovanna Masala, Elisabete Weiderpass, María- Dolores Chirlaque, Roel C. H. Vermeulen, Ruth C. Travis, Melissa C. Southey, Roger L. Milne, Demetrius Albanes, Jarmo Virtamo, Stephanie Weinstein, Jacqueline Clavel, Tongzhang Zheng, Theodore R. Holford, Danylo J. Villano, Ann Maria, John J. Spinelli, Randy D. Gascoyne, Joseph M. Connors, Kimberly A. Bertrand, Edward Giovannucci, Peter Kraft, Anne Kricker, Jenny Turner, Maria Grazia Ennas, Giovanni M. Ferri, Lucia Miligi, Liming Liang, Baoshan Ma, Jinyan Huang, Simon Crouch, Ju-Hyun Park, Nilanjan Chatterjee, Kari E. North, John A. Snowden, Josh Wright, Joseph F. Fraumeni, Kenneth Offit, Xifeng Wu, Silvia de Sanjose, James R. Cerhan, Stephen J. Chanock, Nathaniel Rothman, Susan L. Slager

**Affiliations:** 1 Division of Cancer Epidemiology and Genetics, National Cancer Institute, Bethesda, Maryland 20892, USA; 2 Division of Hematology and Hematologic Malignancies, Department of Internal Medicine, Huntsman Cancer Institute and University of Utah School of Medicine, Salt Lake City, Utah 84112, USA; 3 Department of Epidemiology, School of Public Health and Comprehensive Cancer Center, University of Alabama at Birmingham, Birmingham, Alabama 35233, USA; 4 Division of Environmental Health Sciences, University of California Berkeley School of Public Health, Berkeley, California 94720, USA; 5 Department of Medicine, Memorial Sloan Kettering Cancer Center, New York, New York 10065, USA; 6 Cancer Genomics Research Laboratory, Division of Cancer Epidemiology and Genetics, National Cancer Institute, Gaithersburg, Maryland 20877, USA; 7 Department of Epidemiology, MD Anderson Cancer Center, Houston, Texas 77030, USA; 8 Center for Chronic Immunodeficiency, University Medical Center Freiburg, Freiburg, 79108 Baden-Württemberg, Germany; 9 Department of Epidemiology, Harvard School of Public Health, Boston, Massachusetts 02115, USA; 10 MRC-PHE Centre for Environment and Health, School of Public Health, Imperial College London, London W2 1PG, UK; 11 Department of Medicine, Solna, Karolinska Institutet, Karolinska University Hospital, Stockholm 17176, Sweden; 12 Epidemiology of Childhood and Adolescent Cancers Group, INSERM, Center of Research in Epidemiology and Statistics Sorbonne Paris Cité (CRESS), F-94807 Paris, France; 13 Université Paris Descartes, 75270 Paris, France; 14 Registre des hémopathies malignes de la Gironde, Institut Bergonié, 33076 Bordeaux Cedex, France; 15 Department of Preventive Medicine, USC Keck School of Medicine, University of Southern California, Los Angeles, California 90033, USA; 16 Norris Comprehensive Cancer Center, USC Keck School of Medicine, University of Southern California, Los Angeles, California 90033, USA; 17 Department of Oncology, University of Sheffield, Sheffield, South Yorkshire S10 1NS, UK; 18 Division of Cancer Etiology, City of Hope Beckman Research Institute, Duarte, California 91030, USA; 19 Epidemiology Research Program, American Cancer Society, Atlanta, Georgia 30303, USA; 20 Departamento de Biologia Geral, Instituto de Ciências Biológicas, Universidade Federal de Minas Gerais, Belo Horizonte 31270-901, Brazil; 21 Genome Sciences Centre, BC Cancer Agency, Vancouver, British Columbia, Canada V5Z1L3; 22 Department of Biomedical Physiology and Kinesiology, Simon Fraser University, Burnaby, British Columbia, Canada V5A1S6; 23 Ontario Health Study, Toronto, Ontario, Canada M5G 0A3; 24 Channing Division of Network Medicine, Department of Medicine, Brigham and Women's Hospital and Harvard Medical School, Boston, Massachusetts 02115, USA; 25 Centre for Big Data Research in Health, University of New South Wales, Sydney, New South Wales 2052, Australia; 26 Department of Public Health, Clinical and Molecular Medicine, University of Cagliari, Monserrato, 09042 Cagliari, Italy; 27 Department of Environmental Health Sciences, Yale School of Public Health, New Haven, Connecticut 06520, USA; 28 Cancer Epidemiology Centre, Cancer Council Victoria, Melbourne, Victoria 3004, Australia; 29 Centre for Epidemiology and Biostatistics, Melbourne School of Population and Global Health, University of Melbourne, Melbourne, Victoria 3010, Australia; 30 Department of Population Health, New York University School of Medicine, New York, New York 10016, USA; 31 Department of Environmental Medicine, New York University School of Medicine, New York, New York 10016, USA; 32 Perlmutter Cancer Center, NYU Langone Medical Center, New York, New York 10016, USA; 33 Westat, Rockville, Maryland 20850, USA; 34 Division of Hematology, Mayo Clinic, Rochester, Minnesota 55905, USA; 35 Department of Medicine, Mayo Clinic, Rochester, Minnesota 55905, USA; 36 Department of Laboratory Medicine and Pathology, Mayo Clinic, Rochester, Minnesota 55905, USA; 37 Department of Health Sciences Research, Mayo Clinic, Rochester, Minnesota 55905, USA; 38 Department of Epidemiology Research, Division of Health Surveillance and Research, Statens Serum Institut, 2300 Copenhagen, Denmark; 39 Department of Medical Epidemiology and Biostatistics, Karolinska Institutet, 17177 Stockholm, Sweden; 40 Department of Medicine, Stanford University School of Medicine, Stanford, California 94305, USA; 41 Department of Immunology, Genetics and Pathology, Uppsala University, 75105 Uppsala, Sweden; 42 Center for Epidemiology and Computational Biology, Health Sciences, Exponent, Inc., Menlo Park, California 94025, USA; 43 Division of Epidemiology, Department of Health Research and Policy, Stanford University School of Medicine, Stanford, California 94305, USA; 44 Department of Internal Medicine, Huntsman Cancer Institute, Salt Lake City, Utah 84112, USA; 45 Department of Internal Medicine, University of Utah School of Medicine, Salt Lake City, Utah 84108, USA; 46 George E. Wahlen Department of Veterans Affairs Medical Center, Salt Lake City, Utah 84148, USA; 47 Department of Internal Medicine, Carver College of Medicine, The University of Iowa, Iowa City, Iowa 52242, USA; 48 Department of Epidemiology and Biostatistics, University of California San Francisco, San Francisco, California 94118, USA; 49 Department of Biostatistics, University of Alabama at Birmingham, Birmingham, Alabama 35233, USA; 50 Division of Endocrinology, Diabetes and Metabolism, The Ohio State University, Columbus, Ohio 43210, USA; 51 Division of Public Health Sciences, Fred Hutchinson Cancer Research Center, Seattle, Washington 98117, USA; 52 Cancer Epidemiology Research Programme, Catalan Institute of Oncology-IDIBELL, L'Hospitalet de Llobregat, Barcelona 08908, Spain; 53 CIBER de Epidemiología y Salud Pública (CIBERESP), 08036 Barcelona, Spain; 54 Unit of Nutrition, Environment and Cancer, Cancer Epidemiology Research Program, Catalan Institute of Oncology-IDIBELL, L'Hospitalet de Llobregat, 08908 Barcelona, Spain; 55 Translational Research Laboratory, Catalan Institute of Oncology-IDIBELL, L'Hospitalet de Llobregat, 08908 Barcelona, Spain; 56 Unit of Infections and Cancer (UNIC), Cancer Epidemiology Research Programme, Institut Catala d'Oncologia, IDIBELL, 08908L'Hospitalet de Llobregat, 08908 Barcelona, Spain; 57 CIBER Epidemiología y Salud Pública (CIBERESP), 28029 Madrid, Spain; 58 Division of Cancer Epidemiology, German Cancer Research Center (DKFZ), Heidelberg, 69120 Baden-Württemberg, Germany; 59 The Tisch Cancer Institute, Icahn School of Medicine at Mount Sinai, New York, New York 10029, USA; 60 International Agency for Research on Cancer (IARC), 69372 Lyon, France; 61 Department of Cancer Epidemiology and Genetics, Masaryk Memorial Cancer Institute and MF MU, 656 53 Brno, Czech Republic; 62 EA 4184, Registre des Hémopathies Malignes de Côte d'Or, University of Burgundy and Dijon University Hospital, 21070 Dijon, France; 63 School of Nursing and Human Sciences, Dublin City University, Dublin 9, Ireland; 64 Division of Hematology/Oncology, Mayo Clinic, Phoenix, Arizona 85054, USA; 65 Department of Medicine, Duke University and VA Medical Centers, Durham, North Carolina 27710, USA; 66 Department of Environmental and Occupational Health, Drexel University School of Public Health, Philadelphia, Pennsylvania 19104, USA; 67 Department of Family Medicine and Public Health Sciences, Wayne State University, Detroit, Michigan 48201, USA; 68 School of Public Health, Imperial College London, London W2 1PG, UK; 69 Human Genetics Foundation, 10126 Turin, Italy; 70 Molecular and Nutritional Epidemiology Unit, Cancer Research and Prevention Institute (ISPO), 50139 Florence, Italy; 71 Department of Community Medicine, Faculty of Health Sciences, University of Tromsø, The Arctic University of Norway, N-9037 Tromsø, Norway; 72 Department of Research, Cancer Registry of Norway, Institute of Population-Based Cancer Research, N-0304 Oslo, Norway; 73 Genetic Epidemiology Group, Folkhälsan Research Center, FI-00250 Helsinki, Finland; 74 Department of Epidemiology, Murcia Regional Health Authority, E30008 Murcia, Spain; 75 Institute for Risk Assessment Sciences, Utrecht University, Utrecht, 3508, TD, The Netherlands; 76 Julius Center for Health Sciences and Primary Care, University Medical Center Utrecht, 3584 CX Utrecht, The Netherlands; 77 Cancer Epidemiology Unit, University of Oxford, Oxford OX3 7LF, UK; 78 Genetic Epidemiology Laboratory, Department of Pathology, University of Melbourne, Melbourne, Victoria 3010, Australia; 79 Chronic Disease Prevention Unit, National Institute for Health and Welfare, FI-00271 Helsinki, Finland; 80 Department of Biostatistics, Yale School of Public Health, New Haven, Connecticut 06520, USA; 81 Cancer Control Research, BC Cancer Agency, Vancouver, British Columbia, Canada V5Z1L3; 82 School of Population and Public Health, University of British Columbia, Vancouver, British Columbia, Canada V6T1Z3; 83 Center for Lymphoid Cancer, BC Cancer Agency, Vancouver, British Columbia, Canada V5Z1L3; 84 Department of Pathology, University of British Columbia, Vancouver, British Columbia, Canada V6T1Z3; 85 Department of Medicine, University of British Columbia, Vancouver, British Columbia, Canada V6T1Z3; 86 Department of Nutrition, Harvard School of Public Health, Boston, Massachusetts 02115, USA; 87 Department of Biostatistics, Harvard School of Public Health, Boston, Massachusetts 02115, USA; 88 Sydney School of Public Health, The University of Sydney, Sydney, New South Wales 2006, Australia; 89 Faculty of Medicine and Health Sciences, Macquarie University, Sydney, New South Wales 2109, Australia; 90 Department of Histopathology, Douglass Hanly Moir Pathology, Sydney, New South Wales 2113, Australia; 91 Department of Biomedical Science, University of Cagliari, Monserrato, 09042 Cagliari, Italy; 92 Interdisciplinary Department of Medicine, University of Bari, 70124 Bari, Italy; 93 Environmental and Occupational Epidemiology Unit, Cancer Prevention and Research Institute (ISPO), 50139 Florence, Italy; 94 College of Information Science and Technology, Dalian Maritime University, Dalian, Liaoning Province 116026, China; 95 Department of Health Sciences, University of York, York YO10 5DD, UK; 96 Department of Statistics, Dongguk University, Seoul 100-715, Republic of Korea; 97 Department of Epidemiology, University of North Carolina at Chapel Hill, Chapel Hill, North Carolina 27599, USA; 98 Carolina Center for Genome Sciences, University of North Carolina at Chapel Hill, Chapel Hill, North Carolina 27599, USA; 99 Department of Haematology, Sheffield Teaching Hospitals NHS Foundation Trust, Royal Hallamshire Hospital, Sheffield, South Yorkshire S10 2TN, UK

## Abstract

Chronic lymphocytic leukemia (CLL) is a common lymphoid malignancy with strong heritability. To further understand the genetic susceptibility for CLL and identify common loci associated with risk, we conducted a meta-analysis of four genome-wide association studies (GWAS) composed of 3,100 cases and 7,667 controls with follow-up replication in 1,958 cases and 5,530 controls. Here we report three new loci at 3p24.1 (rs9880772, *EOMES*, *P*=2.55 × 10^−11^), 6p25.2 (rs73718779, *SERPINB6*, *P*=1.97 × 10^−8^) and 3q28 (rs9815073, *LPP*, *P*=3.62 × 10^−8^), as well as a new independent SNP at the known 2q13 locus (rs9308731, *BCL2L11*, *P*=1.00 × 10^−11^) in the combined analysis. We find suggestive evidence (*P*<5 × 10^−7^) for two additional new loci at 4q24 (rs10028805, *BANK1*, *P*=7.19 × 10^−8^) and 3p22.2 (rs1274963, *CSRNP1*, *P*=2.12 × 10^−7^). Pathway analyses of new and known CLL loci consistently show a strong role for apoptosis, providing further evidence for the importance of this biological pathway in CLL susceptibility.

## Abstract (web summary)

Chronic lymphocytic leukemia is a highly inheritable cancer. Here the authors conduct a metaanalysis of four genome-wide association studies and identify three novel loci located near *EOMES*, *SERPINB6* and *LPP* associated with risk of this disease.

## Graphical abstract

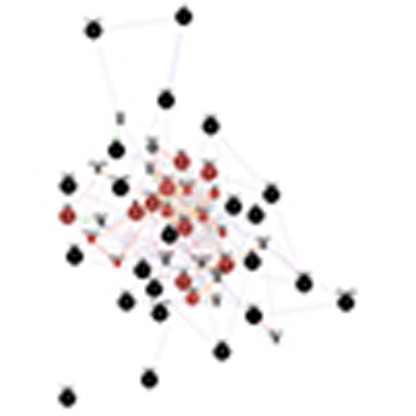

Chronic lymphocytic leukemia (CLL) is the most common leukemia among adults in western countries^1^. Although advances in treatment options have been made, CLL remains an incurable malignancy. Genome-wide association studies (GWAS) have identified multiple susceptibility loci for CLL^2^,^3^,^4^,^5^,^6^,^7^ with at least three loci having more than one independent signal^5^,^8^. However, these discovered loci only account for about a third of the estimated heritability attributed to common variants^5^. In a combined analysis of four GWAS and follow-up replication, including 3,888 cases and 12,539 controls of European ancestry, we recently discovered 11 independent single-nucleotide polymorphisms (SNPs) in nine novel loci associated with CLL risk^5^. To discover additional loci associated with susceptibility to CLL, we more than doubled our replication sample size in the present study, slightly increasing our statistical power, and investigated the association with 14 other promising SNPs identified from our GWAS meta-analysis.

Here, we identify four new independent SNPs in three novel loci as well as two promising new loci associated with the risk of CLL. Pathway analyses with these new loci as well as the previously identified loci suggest a strong role for the apoptosis in susceptibility to CLL, further enhancing our understanding.

## Results

### Discovery meta-analysis

We conducted a meta-analysis of four genome-wide association studies^4^,^5^,^9^ comprising 3,100 unrelated cases and 7,667 controls of European ancestry (see ‘Methods' section, Supplementary Tables 1–3). As these studies used different commercial SNP microarrays, we imputed the ∼8.5 million common SNPs present in the 1000 Genomes Phase 1 integrated data (version 3)^10^ for each study using IMPUTE2 (ref. 11; Supplementary Table 2) and tested for associations with CLL risk assuming a log-additive genetic model. After quality control exclusions, ∼8.5 million SNPs with minor allele frequency >1% were meta-analysed in the discovery stage using a fixed effects model.

A quantile–quantile plot of the meta-analysis results in the discovery stage showed an enrichment of small *P* values from the fixed-effects model compared with the null distribution, which persisted even after removal of the known loci (Supplementary Fig. 1). There was little evidence for inflation due to population stratification (lambda=1.028). Under a log-additive genetic model, a total of 16 unique loci (defined as separated by at least 1 Mb) reached genome-wide significance (*P*<5 × 10^−8^; Supplementary Fig. 2), all of which had been previously reported^2^,^3^,^5^,^8^. For each previously reported locus, we identified the SNP with the strongest *P* value within 1 Mb of the published index SNP. Of the 29 published loci, 21 were at least suggestively associated with CLL under a log-additive model in our discovery meta-analysis with *P*<5 × 10^−7^ (Supplementary Table 4). As the original reported SNPs at two loci (4q26 and 6q25.2) failed to show nominal significance (*P*<0.05) in our study, we meta-analysed our results with the published results for known loci from two other GWAS^6^,^7^. In this larger meta-analysis, 25 of the published loci were at least suggestively associated with CLL risk (*P*<5 × 10^−7^) based on a fixed-effects model; however, both rs6858698 at 4q26 and rs11631963 at 15q25.2 showed attenuated odds ratios and weak *P* values even with this increased sample size (*P*=0.002 and *P*=0.0003, respectively; Supplementary Table 5), questioning the certainty of these loci.

### Joint meta-analysis of the discovery and replication

**Figure 1:**
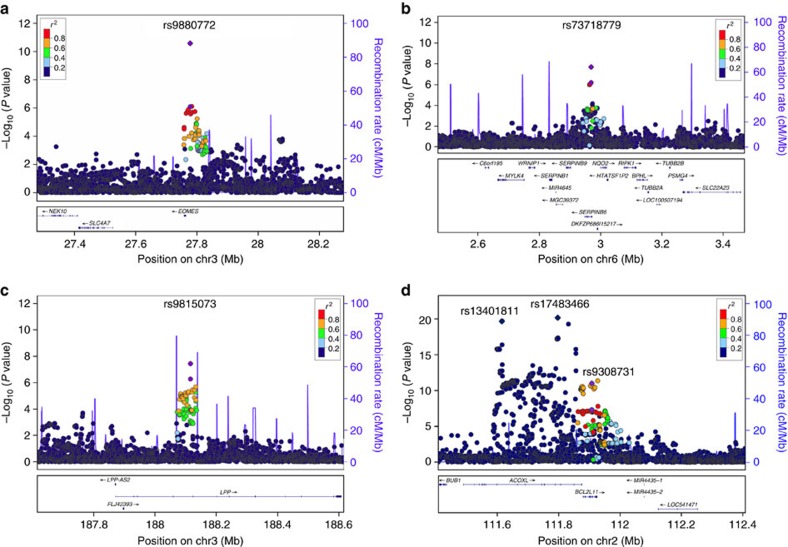
Regional association plots of the three novel loci and new independent SNP at a known locus associated with the risk of CLL. (**a**) Chromosome 3p24.1 (rs9880772), (**b**) chromosome 6p25.2 (rs73718779), (**c**) chromosome 3q28 (rs9815073) and (**d**) chromosome 2q13 (rs9308731). Shown are the −log_10_ association *P* values from the discovery fixed effects meta-analysis (dots) and combined discovery and replication fixed effects meta-analysis (diamonds). The lead SNPs are shown in purple. Estimated recombination rates (from 1000 Genomes) are plotted in blue. The SNPs surrounding the most significant SNP are colour-coded to reflect their correlation with this SNP. Pairwise *r*^2^ values are from 1000 Genomes European data (March 2012 release). Genes, position of exons and direction of transcription from UCSC genome browser (genome.ucsc.edu) are noted. Plots were generated using LocusZoom (http://csg.sph.umich.edu/locuszoom).

To identify additional loci associated with CLL risk, four SNPs in known regions that appeared to be possible secondary signals (*r*^2^<0.1 with the reported SNPs and *P*<5 × 10^−7^ in the discovery meta-analysis) and 10 SNPs in novel regions that reached a significance threshold of *P*<5 × 10^−6^ in the discovery meta-analysis were taken forward for replication in 1,958 cases and 5,530 controls. In the joint meta-analysis of the discovery and replication, four SNPs were identified as genome-wide significant under a fixed-effects model, three in novel regions and one as a new independent SNP in the previously reported 2q13 region: 3p24.1 (rs9880772, *EOMES*, *P*=2.55 × 10^−11^), 6p25.2 (rs73718779, *SERPINB6*, *P*=1.97 × 10^−8^), 3q28 (rs9815073, *LPP*, *P*=3.62 × 10^−8^) and 2q13 (rs9308731, *BCL2L11*, *P*=1.00 × 10^−11^; Table 1, Fig. 1, Supplementary Table 6). The new 2q13 SNP, rs9308731, was weakly correlated with the two previously identified^2^,^5^ independent SNPs at 2q13, rs17483466 (*r*^2^=0.008) and rs13401811 (*r*^2^=0.0005); when the three 2q13 SNPs were included in the same logistic regression model, all three remained genome-wide significant (Supplementary Table 7). Genome-wide suggestive evidence (*P*<5 × 10^−7^) was also found in the joint discovery/replication fixed-effects meta-analysis for two promising novel loci at 4q24 (rs10028805, *BANK1*, *P*=7.19 × 10^−8^) and 3p22.2 (rs1274963, *CSRNP1*, *P*=2.12 × 10^−7^; Table 1, Supplementary Fig. 3).

## Discussion

All the three novel loci are located in or near genes implicated in apoptosis and/or immune function. The novel 3p24.1 SNP (rs9880772) resides 13 kb 5′ of eomesodermin (*EOMES*), a member of the T-box gene family and a key regulator in cell-mediated immunity and CD8+ T-cell differentiation^12^. *EOMES* is critical for lymphoproliferation due to Fas-deficiency^13^, which has been observed in inherited lymphoproliferative disorders associated with autoimmunity^14^,^15^. Overexpression of *EOMES* has been observed among extranodal natural killer/T (NK/T)-cell and peripheral T-cell lymphomas^16^. Interestingly, highly correlated SNPs within the same 15 kb region 5′ of *EOMES* have also been associated with two autoimmune diseases, rheumatoid arthritis^17^ (rs3806624, *r*^2^=0.96) and multiple sclerosis^18^ (rs11129295, *r*^2^=0.72), as well as Hodgkin's lymphoma^19^ (rs3806624, *r*^2^=0.96), underscoring the importance of this genetic region for susceptibility to both lymphoma and autoimmune disease. Regions locally centromeric and telomeric of rs9880772 show strong regulation and promoter signatures by histone marks, DNaseI hypersensitivity and transcription factor binding sites, and the correlated SNP, rs3806624, is located within a poised promoter in the lymphoblastoid cell line, GM12878 (Supplementary Table 8).

The novel 6p25.2 SNP (rs73718779) is located within an intron of *SERPINB6*, which encodes a member of the serine protease inhibitor (serpin) superfamily. Although the physiological role of *SERPINB6* is not well understood, it inhibits cathepsin G^20^, which activates the pro-apoptotic proteinase caspase 7 (ref. 21). In eQTL and methylation QTL analyses, we found that the T allele for rs6939693, an SNP completely correlated with rs73718779 (*r*^2^=1), was associated with significantly reduced *SERPINB6* expression in blood in a weighted z-score meta-analysis (*P*=1.40 × 10^−52^, Supplementary Table 9) and increased DNA methylation levels based on a linear mixed model (*P*=1.70 × 10^−11^, Supplementary Table 10), suggesting strong potential functional relevance.

The 3q28 SNP (rs9815073) is an intronic variant within the LIM domain containing preferred translocation partner in lipoma gene (*LPP*). The SNP is located within a strong enhancer in the lymphoblastoid cell line, GM12878 (Supplementary Fig. 4). Moderately correlated SNPs in *LPP* have previously been associated with diseases related to autoimmunity and/or immune dysregulation, including celiac disease^22^ (rs1464510, *r*^2^=0.51), allergy^23^ (rs9860547, *r*^2^=0.68) and vitiligo^24^ (rs1464510, *r*^2^=0.51). SNPs within this region have also been associated with follicular lymphoma^25^ (rs6444305, *r*^2^=0.001) and B-cell lymphoma in Asians (rs6773854, *r*^2^=0.002); however, the association with rs9815073 appears to be independent of both of these SNPs in the fixed-effects meta-analysis (*P*_rs9815073_=9.11 × 10^−7^ after conditioning on rs6444305 and *P*_rs9815073_=5.11 × 10^−7^ after conditioning on rs6773854 compared with *P*_rs9815073_=5.35 × 10^−7^ without adjustment).

The suggestive 4q24 SNP (rs10028805) is located within an intron of B-cell scaffold protein with ankyrin repeats 1 (*BANK1*), which encodes a protein adaptor that is predominantly expressed in B-cells. *BANK1* is a putative tumour suppressor gene in B-cell lymphomagenesis^26^, and *BANK1*-deficient cells show enhanced CD40-mediated proliferation and survival with Akt activation^27^. Rs10028805 is moderately correlated with rs10516487 (*r*^2^=0.70), a non-synonymous SNP in exon 2 that has been associated with systemic lupus erythematosus^28^ and shown to alter mRNA splicing and the quantity of the *BANK1* protein^29^. Consistent with this, we observed rs10028805 to be associated with *BANK1* expression in lymphoblastoid cells (*P*=6.89 × 10^−13^, Supplementary Table 11).

The 3p22.2 SNP (rs1274963) is an intronic variant in the gene *CSRNP1* (cysteine-serine-rich nuclear protein 1), which is induced by AXIN1, a scaffold protein that is a negative regulator of the Wnt/signalling pathway^30^. A putative tumour suppressor with potential apoptosis activity^31^, *CSRNP1* plays an important role in the development of haematopoiesis progenitors in zebrafish^32^ and has been shown to be expressed in many tissues, with leukocytes being among those with the highest abundance^30^. The SNP resides in an area with strong regulatory potential based on histone marks, DNaseI hypersensitivity and transcription factor binding sites (Supplementary Table 8) and is located within a strong enhancer in the lymphoblastoid cell line, GM12878 (Supplementary Fig. 4). Of potential functional relevance, in lymphocytes and blood, the rs1274963A risk allele was associated with reduced *WDR48* expression (Supplementary Tables 9 and 11), a gene shown to induce apoptosis and suppress tumour cell proliferation^33^.

**Figure 2:**
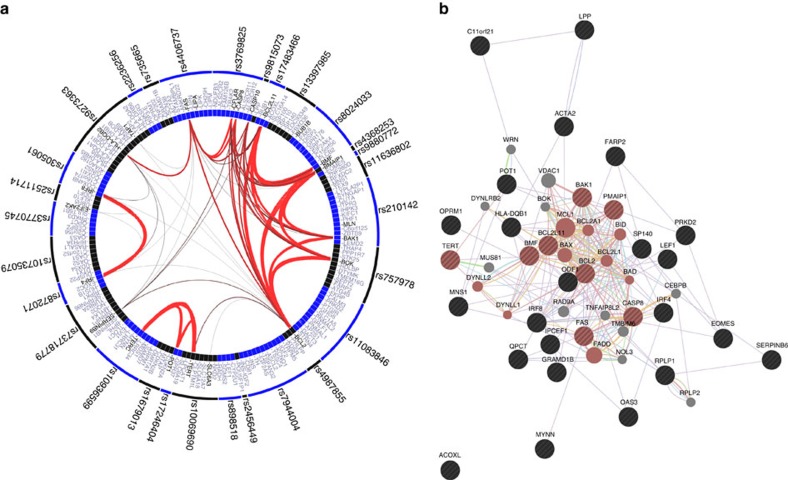
Relationships between loci associated with CLL risk. (**a**) The GRAIL results are depicted in a circle plot with the connections between the SNPs and corresponding gene for the established CLL loci. The width of the line corresponds to the strength of the literature-based connectivity with thicker lines representing stronger connections. (**b**) Depiction of GeneMania results. Query genes are shown in large circles with hatch marks and tightly connected neighbouring genes were shown in small solid circles. The genes belonging to the top function, ‘regulation of apoptotic signalling pathway', are highlighted with red colour. The colour of the line indicates the network: co-expression (lavender), co-localization (purple), genetic interactions (grey), pathway (blue), physical interactions (pink), predicted (orange) and shared protein domains (beige). The figure was created with GeneMania Application version: 3.1.2.8.

To explore potential biological pathways associated with the newly discovered loci as well as the previously established loci for CLL, we conducted pathway analyses using GRAIL^34^, Webgestalt and GeneMania (see ‘Methods' section). All the three pathway analyses identified apoptosis or apoptosis-related pathways as either the top key words (GRAIL, Supplementary Table 12, Fig. 2a) or their most significantly enriched pathway: regulation of apoptotic signalling (GeneMania, *P*=2.06 × 10^−17^, false discovery rate-corrected hypergeometric test, Supplementary Table 13, Fig. 2b) and activation of pro-apoptotic gene products (Webgestalt, *P*=5.49 × 10^−11^, false discovery rate-corrected hypergeometric test, Supplementary Table 14). Other enriched pathways included related apoptotic functions and pathways, such as cytochrome *c* release from mitochondria (Webgestalt, *P*=2.16 × 10^−6^; GeneMania, *P*=7.50 × 10^−13^) and mitochondrial outer membrane (Webgestalt, *P*=3.89 × 10^−6^; GeneMania, *P*=7.18 × 10^−17^; Supplementary Tables 13 and 14, Supplementary Fig. 5). Lymphocyte-related pathways, such as lymphocyte homeostasis (Webgestalt, *P*=2.16 × 10^−6^), haematopoietic or lymphoid organ development (GeneMania, *P*=0.009), and lymphoid (GRAIL) were also observed in all the three analyses.

We constructed a polygenetic risk score that included the four new SNPs from this study as well as 30 previously identified SNPs at known loci (Supplementary Table 5) to evaluate the possibility of risk stratification for CLL (see ‘Methods' section). Those in the top 20% of the risk distribution had a 1.9-fold increased risk (95% confidence interval: 1.70–2.21) compared with those in the middle quintile of the distribution. The newly discovered SNPs explain ∼1% of the familial risk. Together with the previously identified loci, we estimate that the identified loci for CLL thus far explain ∼16.5% of the familial risk, which is similar to previous estimates^5^,^6^.

In conclusion, our meta-analysis of GWAS identified four new independent SNPs and two additional promising loci for CLL, furthering our knowledge of the underpinnings of genetic susceptibility to CLL. Pathway analyses of known and new CLL loci point to regulation of apoptosis as one of the key biological processes underlying the genetic loci to date and suggest new avenues for disease prevention and treatment.

## Methods

### Discovery meta-analysis

Our discovery meta-analysis included four CLL GWAS of European ancestry: National Cancer Institute NHL GWAS (NCI GWAS)^5^, Utah Chronic Lymphocytic Leukemia GWAS (UTAH), Genetic Epidemiology of CLL Consortium GWAS (GEC)^4^, and Molecular Epidemiology of Non-Hodgkin Lymphoma GWAS (UCSF)^9^. Details of the case and control ascertainment and study design of the four GWAS, including the 22 studies that comprise the NCI GWAS, are described in Supplementary Table 1. In brief, CLL cases were ascertained from cancer registries, clinics or hospitals, or through self-report verified by medical and pathology reports. For the NCI GWAS, phenotype information for the cases was reviewed centrally at the International Lymphoma Epidemiology Consortium (InterLymph) Data Coordinating Center and harmonized according to the hierarchical classification proposed by the Interlymph Pathology Working Group based on the World Health Organization classification (2008)^35^,^36^. All the studies obtained informed consent from their participants and approval from their respective Institutional Review Boards for this study^5^.

To maximize our statistic power, all cases with sufficient DNA and a subset of available controls were genotyped for this study. Subjects in these studies were genotyped using the Illumina OmniExpress, Omni2.5, HumanHap610K, HumanCNV360-Duo or Affymetrix 6.0. For the NCI GWAS, the majority of subjects were genotyped with the Illumina OmniExpress; however, a subset of controls (*N*=3,536) and one case were genotyped using the Omni2.5, so to prevent potential platform artifacts, extensive quality control metrics were used, including the removal of assays with low completion rates or monomorphic calls from either platform, before combining the data^5^. For all four GWAS, rigorous quality control metrics were applied to each study to ensure high quality results. Samples with poor call rates, gender discordance, abnormal heterozygosity or of non-European ancestry were excluded, and SNPs with a call rate <95% or Hardy–Weinberg equilibrium *P* value <1 × 10^−6^ were removed from the analysis (Supplementary Table 2).

Each GWAS was imputed separately using IMPUTE2 (ref. 11). In contrast to the previous study^5^ where a hybrid reference panel was used for imputation, all the studies in this analysis were imputed using the 1000 Genomes Project version 3 (March 2012 release) as the reference panel. Poorly imputed SNPs (INFO score <0.3) and SNPs with minor allele frequency <1% were excluded from each study, leaving roughly ∼8.5 million SNPs for analysis. After quality control filters, a total of 3,100 cases and 7,667 controls across the four studies remained for analysis (Supplementary Table 3). For each study, principal component analyses were conducted separately. Association testing was conducted for each study separately using SNPTEST version 2, adjusting for age, sex and significant principal components (*P*<0.05 in null model with age and sex). Meta-analyses were performed using the fixed-effects inverse variance method based on the beta estimates and standard errors from each study.

### Replication and technical validation

Replication of potential novel SNPs was undertaken in 1,958 additional cases and 5,530 controls from six different studies (Supplementary Tables 1 and 3). Fourteen promising SNPs that reached a significance threshold of *P*<5 × 10^−6^ in the discovery meta-analysis were taken forward for replication, including 10 SNPs in novel regions (defined as at least 1 Mb from a known CLL locus) and four SNPs in known regions that appeared to be possible secondary signals (*r*^2^<0.1 with the reported SNPs and *P*<5 × 10^−7^ in the discovery meta-analysis). To conduct conditional analyses with the potential secondary signals, the previously reported index SNP(s) in each of these four regions were also genotyped. TaqMan custom genotyping assays (Applied Biosystems) were designed and optimized for the 14 promising SNPs as well as five previously reported index SNPs. Taqman or Sequenom genotyping was conducted separately for each replication study at their own centre. Each study included duplicates for quality control, and HapMap samples genotyped across the centres yielded excellent concordance (100%). Association testing was conducted separately for each study, adjusting for age, sex and for MSKCC, Ashkenazi ancestry. The replication studies were then meta-analysed together and with the discovery GWAS using an inverse variance fixed effects model. All the SNPs reaching genome-wide or suggestive significance in the joint meta-analysis were either directly genotyped or well imputed (INFO>0.78 for all SNPs with average INFO=0.95) in the GWAS. Technical validation comparing genotype calls or imputed data from the NCI GWAS with Taqman assays for 639 samples revealed moderate concordance for rs9815073 (*r*^2^=0.67), but high concordance (*r*^2^>0.97) for the other SNPs. Although the concordance was lower than expected and further confirmation is needed, an analysis of the Taqman validation data for rs9815073 showed an odds ratio=1.30, which is similar to the odds ratio observed in the full discovery data set.

### Polygenic risk score analysis

To evaluate possible stratification for CLL risk based on the 34 independent SNPs from the 30 loci, we performed a polygenic risk score analysis using the discovery sample data. Polygenic risk scores were derived for each person by taking the weighted sum of the risk alleles (0, 1 or 2) for each of the 34 SNPs. The weights for each SNP were the per-allele log odds ratios estimated from our meta-analysis of the discovery data. We then computed the quintiles of the polygenic risk scores and used logistic regression models to estimate the odds ratio for CLL risk for each quintile with the middle quintile as the reference. Departures from a multiplicative model were assessed by testing for all pair-wise SNP interactions. No evidence of significant interactions was observed.

### Heritability analysis

To estimate the familial risk explained by both the novel and previously established loci for CLL, we estimated the contribution of each independent SNP to the heritability using the equation *h*^2^_SNP_=*β*^2^2*f*(1−*f*), where *β* is the log-odds ratio per copy of the risk allele from the replication stage analyses and *f* is the allele frequency, and summed the contributions of all novel and established SNPs^37^. We then estimated the total heritability from the sibling relative risk (relative risk=8.5 from Goldin *et al.*^38^), using the equation derived by Pharoah *et al.*^39^ We then calculated the proportion of familial risk explained by dividing the summed contributions of the novel and established SNPs by the total heritability.

### Expression quantitative trait loci and other related analyses

To explore the potential functional relevance of the CLL-associated SNPs, we conducted expression quantitative trait loci (eQTL) and methylation quantitative trait loci (meQTL) analyses using three independent data sets: (1) a childhood asthma study of gene expression in lymphoblastoid cell lines^40^, (2) a meta-analysis of eQTL associations from whole blood^41^, and (3) meQTL in CD4+ lymphocytes from the GOLDN study^42^. In the childhood asthma study^40^, RNA was extracted from lymphoblastoid cell lines from 830 parents and offspring from 206 families of European ancestry. Gene expression was assessed with the Affymetrix HG-U133 Plus 2.0 chip, and subjects were genotyped using the Illumina Human-1 and HumanHap300K beadchips with subsequent imputation using data from the 1000 Genomes Project. The four new and two suggestive SNPs were tested for *cis* associations (defined as gene transcripts within 1 Mb), adjusting for non-genetic effects in the gene expression value and relatedness using MERLIN^43^. To gain insight into the relative importance of associations with our SNPs compared with other SNPs in the region, conditional analyses were also conducted, in which both the CLL SNP and the most significant SNP for the particular gene transcript (that is, the peak SNP) were included in the same model. The meta-analysis of eQTL associations from whole blood^41^ included eQTL data generated using Illumina gene expression arrays from seven studies consisting of a total of 5,311 unrelated Europeans. Gene expression arrays were harmonized by matching probe sequences, and all the studies were imputed using the HapMap European reference panel. SNPs that were strongly correlated (*r*^2^>0.8) with the newly discovered and suggestive CLL SNPs were examined for possible *cis* associations. In the GOLDN study^42^, over 450,000 CpG methylation sites were genotyped in CD4+ T-cells from 593 participants. Subjects were genotyped with the Affymetrix Human SNP Array 6.0, and the 2.5 million SNPs available in the HapMap2 release were imputed. We updated the analysis by including more participants (*n*=717) and expanded the scope of *cis*-meQTL to SNPs and CpG sites within 50 kb of each other. The association between the CLL-associated SNPs (as well as strongly correlated SNPs, *r*^2^>0.8) and methylation beta values was tested using the linear mixed models, adjusting for family structure and other covariates including age, sex, recruitment centres and principal components. Finally, we also utilized HaploReg^44^, a tool for exploring noncoding functional annotation using ENCODE data, to evaluate the genome surrounding our SNPs.

### Pathway analyses

To explore potential biological pathways underlying known CLL loci to date, we conducted analyses using GRAIL^34^, Webgestalt^45^ and GeneMania^46^. GRAIL^34^ is a text-based mining tool that is used to evaluate the relationship between genes at different disease loci. Genes within 250 kb of known loci were included, and the 2006 text database was used to avoid overweighting the previously published loci. Webgestalt^45^ is a web-based pathway analysis server offering hypergeometric tests for Gene Ontology (GO) term enrichments and visualization of enriched GO terms in a graph depicting the GO hierarchy. GeneMania^46^ is a network-based analysis server that finds an expanded set of genes including the query genes and additional genes closely linked with the query genes via protein and genetic interactions, pathways, co-expression, co-localization and protein domain similarity. For both Webgestalt and GeneMania, the nearest gene for each locus was included. For all pathways analyses, only newly discovered loci and the previously identified loci that reached at least *P*<1 × 10^−5^ in the combined meta-analysis with the published results from two other GWAS^6^,^7^ (Supplementary Table 5) were included.

### Chromatin state dynamics analysis

To assess chromatin state dynamics, we used Chromos^47^, which utilizes Chip-Seq data from ENCODE^48^ on nine cell types: B-lymphoblastoid cells (GM12878), hepatocellular carcinoma cells (HepG2), embryonic stem cells (hESC), erythrocytic leukemia cells (hK562), umbilical vein endothelial cells (hUVEC), skeletal muscle myoblasts (hSMM), normal lung fibroblasts (hNHLF), normal epidermal keratinocytes (hNHEK) and mammary epithelial cells (hMEC). This programme uses pre-computed data with genome-segmentation performed using a multivariate hidden Markov-model to reduce the combinatorial space to a set of interpretable chromatin states. The output from Chromos lists data into 15 chromatin states corresponding to repressed, poised and active promoters, strong and weak enhancers, putative insulators, transcribed regions and large-scale repressed and inactive domains. For this study, we focused on the results observed for the lymphoblastoid cell line (GM12878).

## Additional information

**How to cite this article:** Berndt, S. I. *et al.* Meta-analysis of genome-wide association studies discovers multiple loci for chronic lymphocytic leukemia. *Nat. Commun.* 7:10933 doi: 10.1038/ncomms10933 (2016).

## Supplementary Material

Supplementary Information Supplementary Figures 1-5 and Supplementary Tables 1-14

## Figures and Tables

**Table 1:** New loci and independent SNPs associated with CLL risk.

**SNP**	**Cytoband**	**Nearest gene**	**Position**	**Stage**	**No. of cases**	**No. of controls**	**Risk allele/other allele**	**RAF**	**OR**	**CI**	***P***
*New loci*											
rs9880772	3p24.1	*EOMES*	27777779	Discovery	3,097	7,664	T/C	0.464	1.17	(1.10–1.24)	7.77E−07
				Replication	1,935	5,414	T/C	0.467	1.23	(1.13–1.34)	4.67E−06
				Combined	5,032	13,078	T/C	0.465	1.19	(1.13–1.25)	2.55E−11
rs73718779	6p25.2	*SERPINB6*	2969278	Discovery	3,097	7,663	A/G	0.111	1.27	(1.16–1.40)	6.22E−07
				Replication	1,871	4,107	A/G	0.109	1.21	(1.05–1.40)	0.008
				Combined	4,968	11,770	A/G	0.110	1.26	(1.16–1.36)	1.97E−08
rs9815073	3q28	*LPP*	188115682	Discovery	3,098	7,663	C/A	0.651	1.20	(1.12–1.28)	5.35E−07
				Replication	1,848	4,094	C/A	0.652	1.13	(1.03–1.25)	0.01
				Combined	4,946	11,757	C/A	0.651	1.18	(1.11–1.25)	3.62E−08
											
*New independent SNP at known locus*											
rs9308731	2q13	*BCL2L11*	111908262	Discovery	3,100	7,665	A/G	0.541	1.19	(1.12–1.26)	4.71E−08
				Replication	1,929	5,448	A/G	0.531	1.21	(1.10–1.32)	4.66E−05
				Combined	5,029	13,113	A/G	0.537	1.19	(1.13–1.26)	1.00E−11
											
*New suggestive loci (*P*<5 × 10^−7^)*											
rs10028805	4q24	*BANK1*	102737250	Discovery	3,099	7,665	G/A	0.625	1.16	(1.09–1.23)	7.04E−06
				Replication	1,876	4,107	G/A	0.621	1.15	(1.05–1.15)	0.003
				Combined	4,975	11,772	G/A	0.624	1.16	(1.10–1.22)	7.19E−08
rs1274963	3p22.2	*CSRNP1*	39191029	Discovery	3,100	7,666	T/C	0.210	1.20	(1.12–1.29)	1.37E−06
				Replication	1,938	5,402	T/C	0.204	1.13	(1.01–1.26)	0.03
				Combined	5,038	13,068	T/C	0.208	1.18	(1.11–1.25)	2.12E−07

CI, confidence interval; OR, odds ratio; RAF, risk allele frequency among controls.
