# Supplementary material for: Meta-analysis of genome-wide association studies discovers multiple loci for chronic lymphocytic leukemia
Source: Nat Commun. 2016 Mar 9;7:10933. doi: 10.1038/ncomms10933 (PMC4786871; doi:10.1038/ncomms10933)
Supplement: Supplementary Information — Supplementary Figures 1-5 and Supplementary Tables 1-14 [file ncomms10933-s1.pdf]

1. SUPPLEMENTARY FIGURES

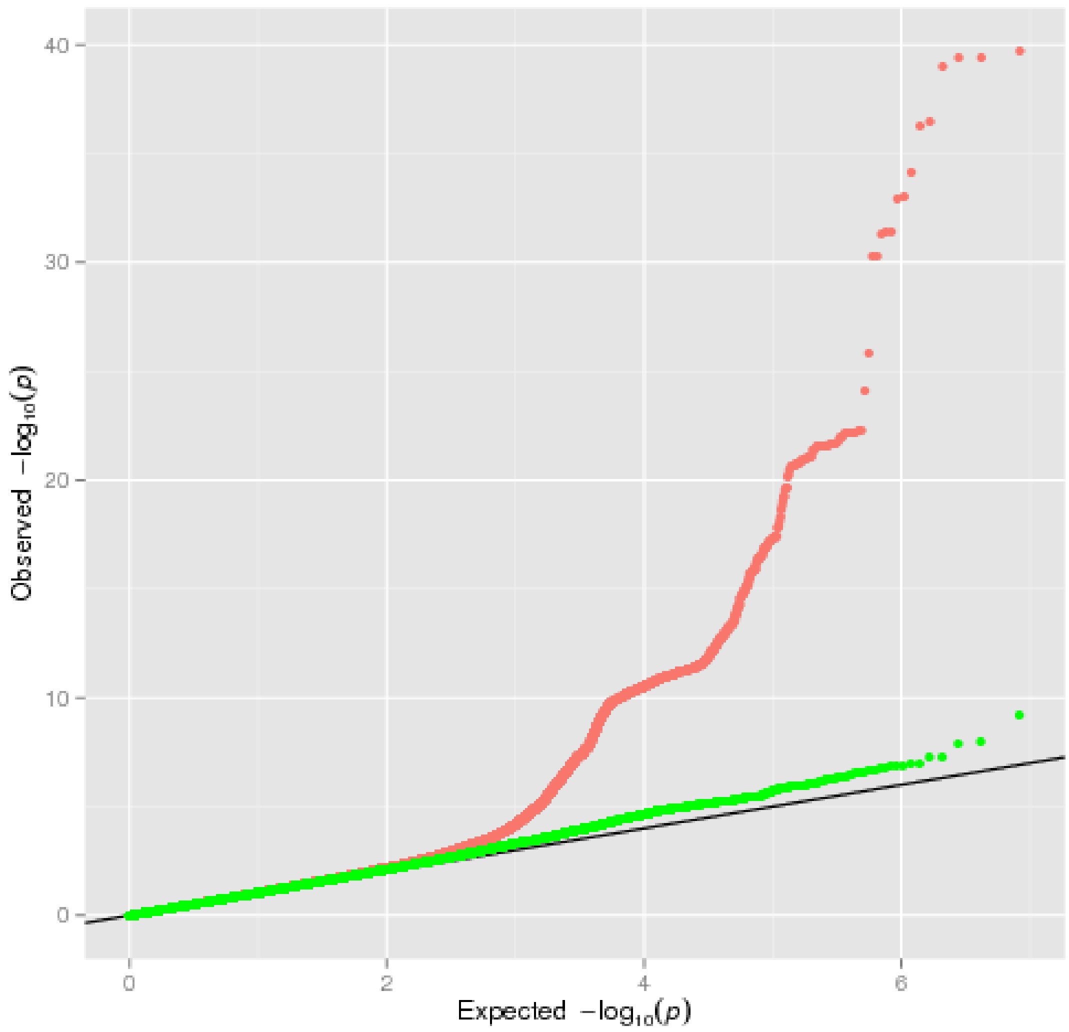

Supplementary Figure 1. Quantile-quantile (Q-Q) plot of the discovery meta-analysis p-values before (red) (lambda=1.028) and after removing any SNPs within 500 kb of a previously established locus (green).

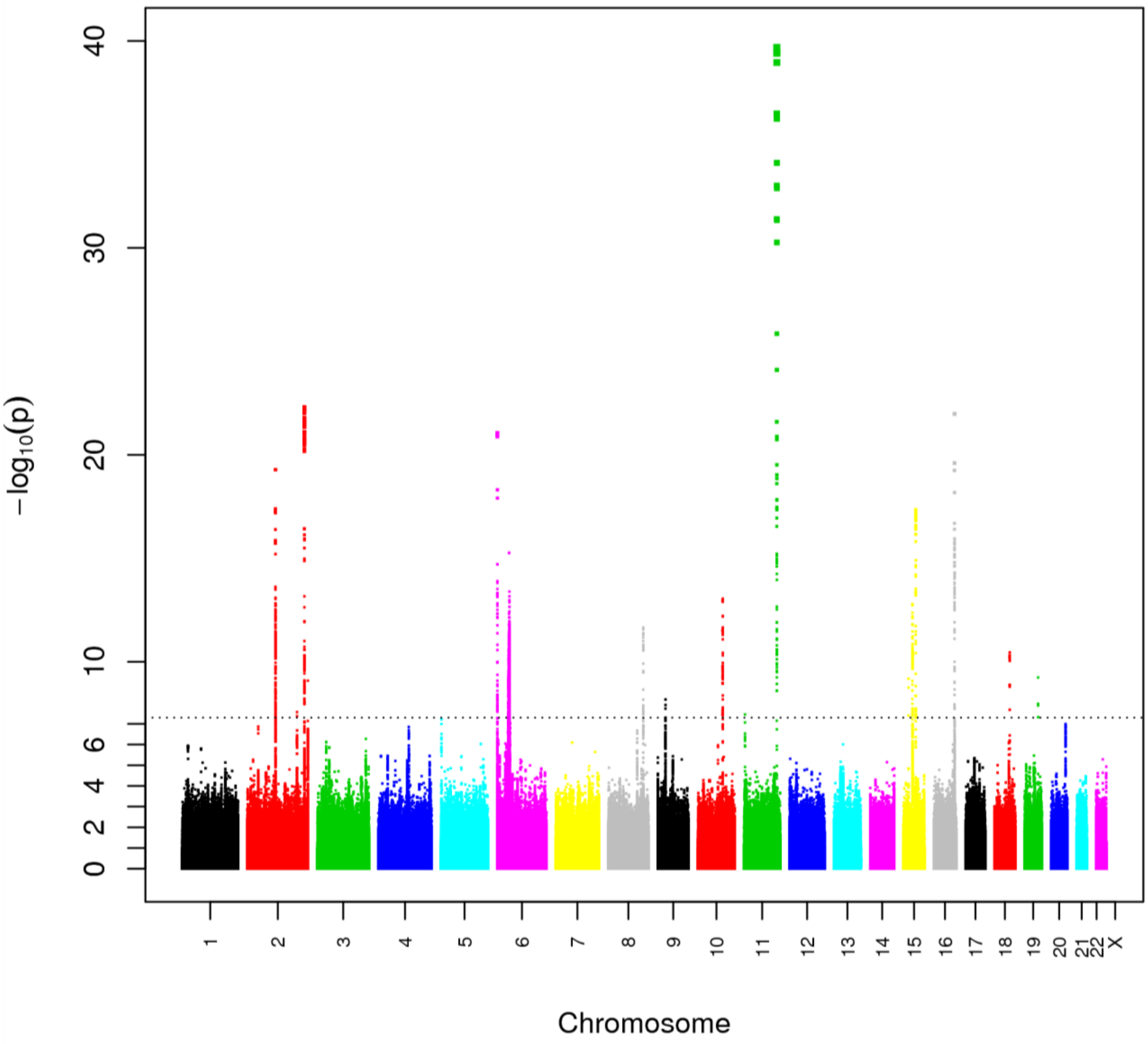

Supplementary Figure 2. Manhattan plot of the discovery meta-analysis –log10 p-values by chromosome position. Each chromosome is plotted with a different color. The dashed horizontal line indicates genome-wide significance ($5x10^{-8}$).

a.

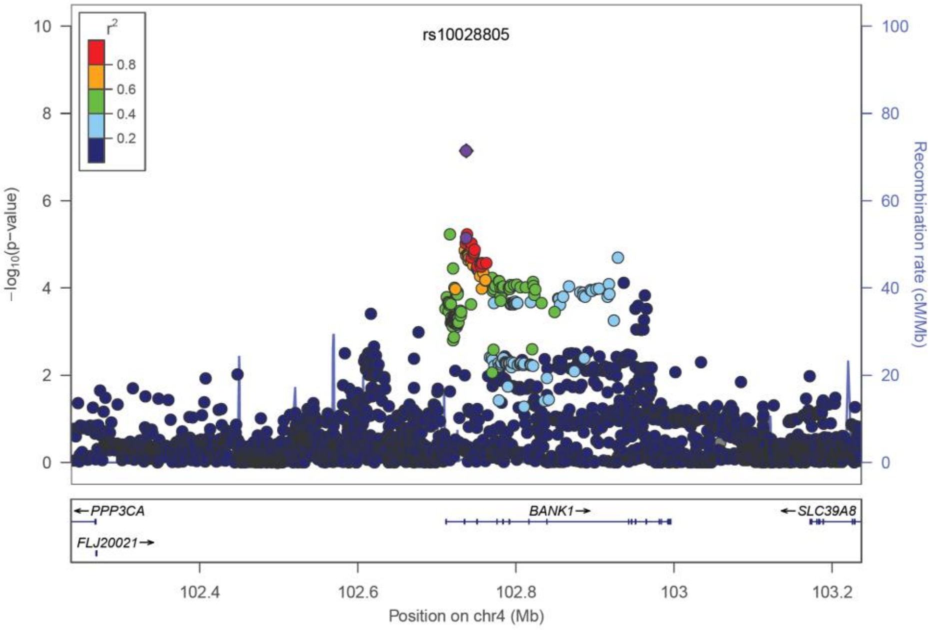

b.

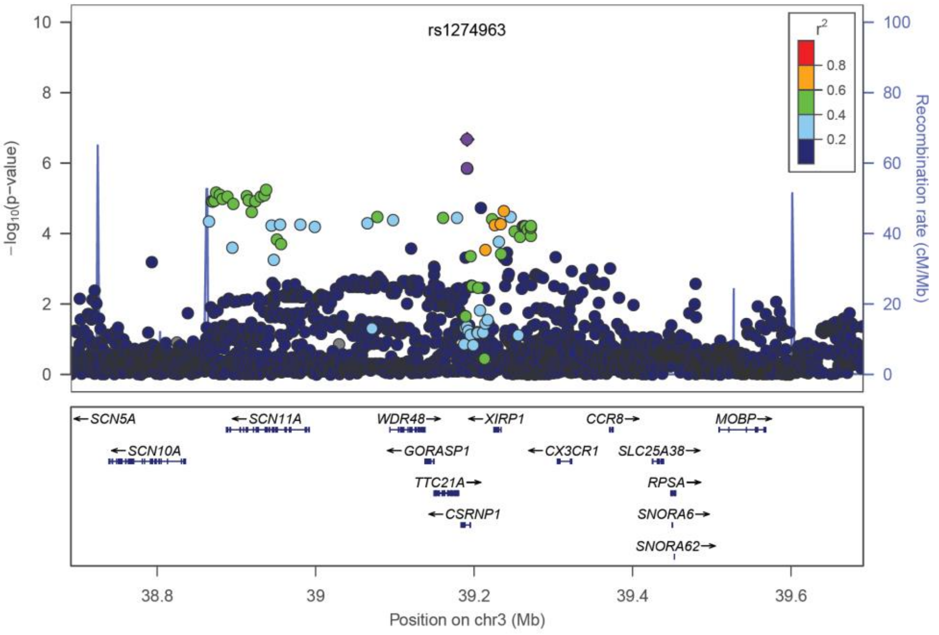

Supplementary Figure 3. Regional plots of the suggestive loci, 4q24 (a) and 3p22.2 (b), are plotted by position on chromosome against the association with CLL (-log P-value) from the discovery fixed effects meta-analysis (dots) and for the lead SNP, the combined discovery and replication fixed effects meta-analysis (purple diamond). The lead SNPs, rs10028805 at 4q24 and rs1274963 at 3p22.2, are shown in purple. Estimated recombination rates (from 1000 Genomes) are plotted in blue. The SNPs surrounding the most significant SNP are color-coded to reflect their correlation with this SNP. Pairwise r^2 values are from 1000 Genomes European data (March 2012 release). Genes, position of exons, and direction of transcription from UCSC genome browser (genome.ucsc.edu) are noted. Plots were generated using LocusZoom (http://csg.sph.umich.edu/locuszoom).

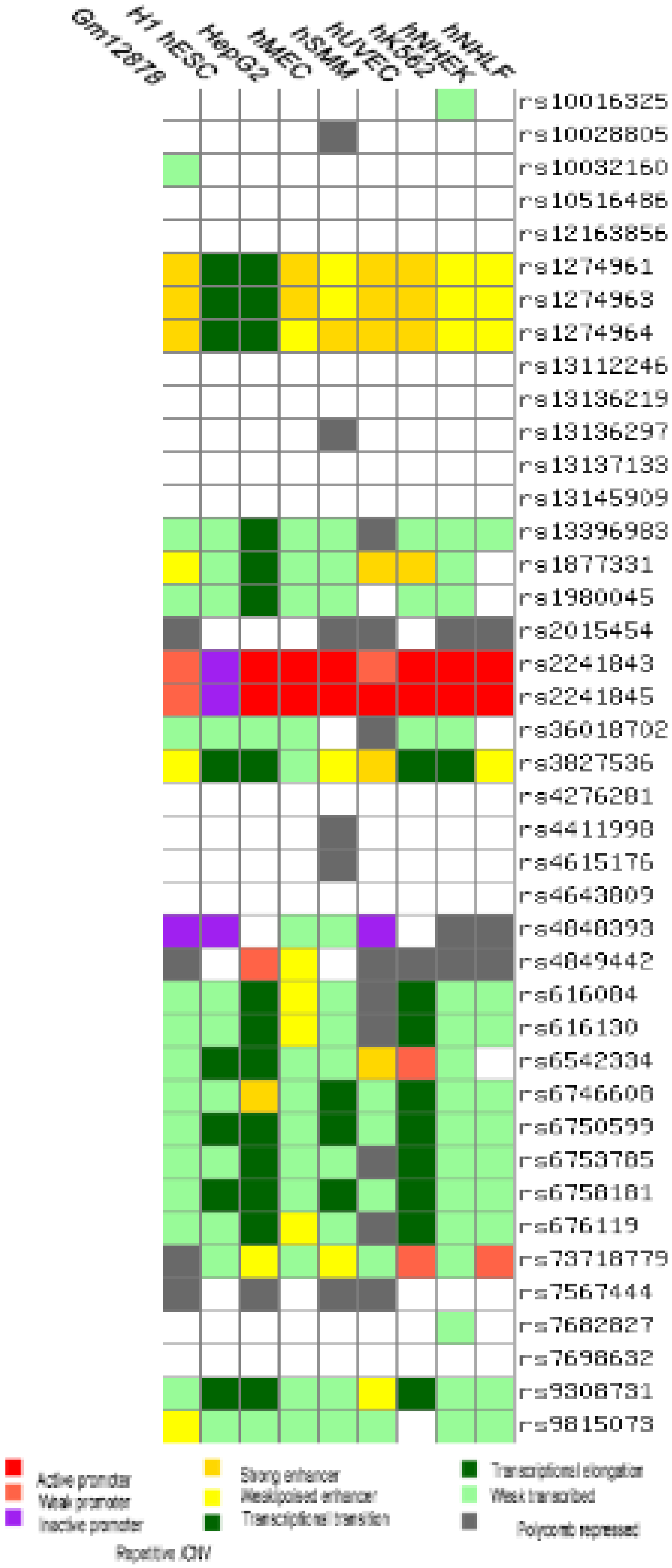

Supplementary Figure 4. Chromatin states at new and suggestive CLL SNPs and proxies (r^2>0.8)

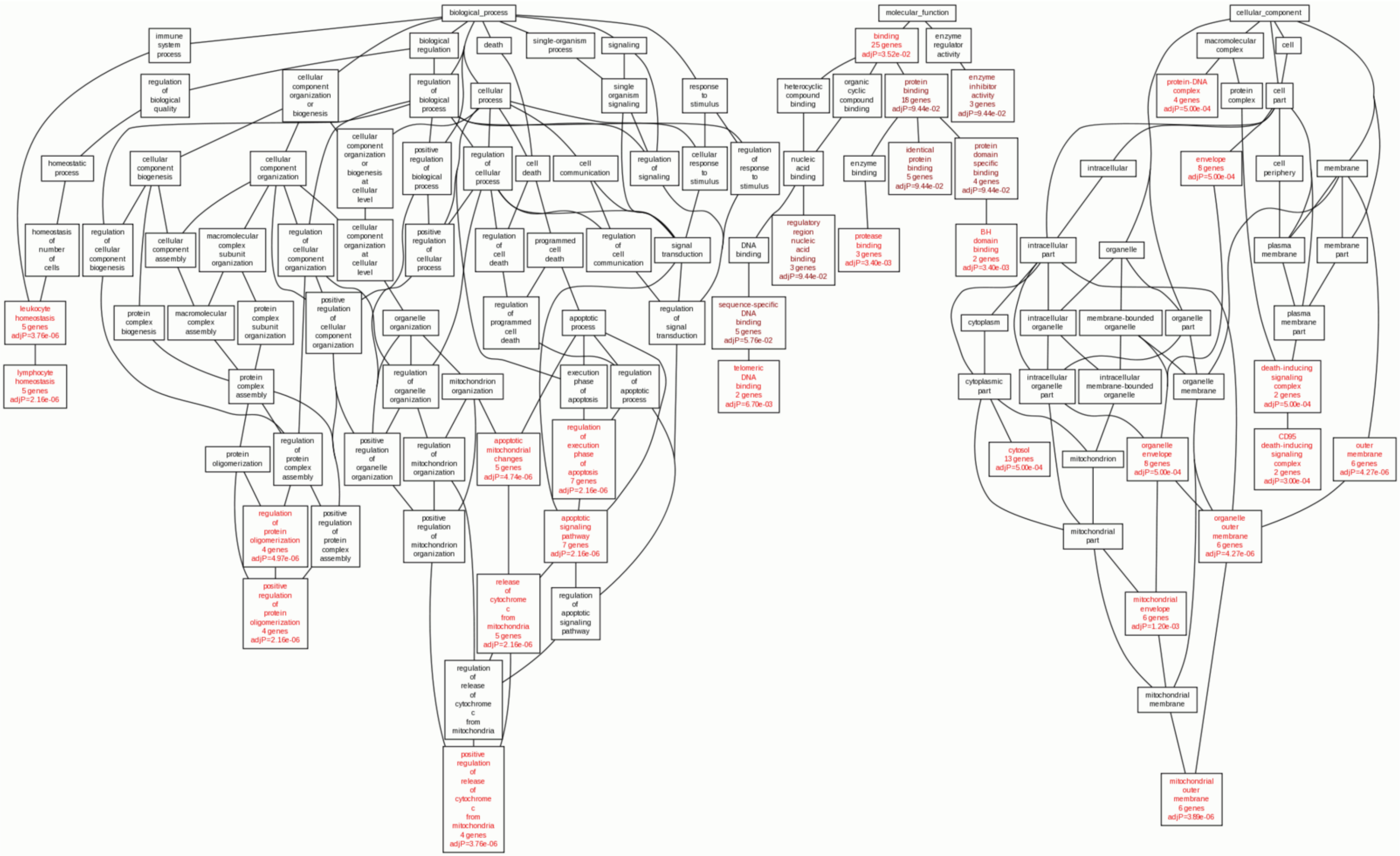

Supplementary Figure 5. Pathways identified by Webgestalt

2. SUPPLEMENTARY TABLES

Supplementary Table 1. Description and study design of studies included in the discovery and replication

Study Name	Study Abbreviation	No. CLL Cases[a]	No. Controls[a]	Design, location	Source of cases	Source of controls	Study Reference
**DISCOVERY – NCI GWAS**							
Alpha-Tocopherol, Beta-Carotene Lung Cancer Prevention Study	ATBC	73	240[b]	Nested case-control, Finland	Identified through linkage to the Finnish Cancer Registry	Cohort participants without a diagnosis of cancer	**[PMID: 8205268]** The alpha-tocopherol, beta-carotene lung cancer prevention study: design, methods, participant characteristics, and compliance. The ATBC Cancer Prevention Study Group. *Ann Epidemiol* 1994 Jan;4(1):1-10.
American Cancer Society Cancer Prevention Study-II Nutrition Cohort	CPS-II	282	220[b]	Nested case-control, USA	Self-report through biannual questionnaires (starting in 1997). Verified by medical records or linkage to state cancer registry	Cohort participants alive at time of case diagnosis without cancer	**[PMID:11900235]** Calle EE. et al. The American Cancer Society Cancer Prevention Study II Nutrition Cohort: rationale, study design, and baseline characteristics. *Cancer* 2002;94:2490-501.
European Prospective Investigation into Cancer, Chronic Diseases, Nutrition and Lifestyles	EPIC	81	773	Nested case-control, multiple European countries	Cases identified through population cancer registries in seven of the participating countries (Denmark, Italy, The Netherlands, Norway, Spain, Sweden and the UK) and through a combination of methods including health insurance records, cancer and pathology registries, and by active follow-up through study subjects and their next-of-kin in three countries (France, Germany and Greece)	Cohort participants matched by age, sex and study center who were alive and cancer-free at the time of diagnosis of the corresponding case	**[PMID:9126529]**Riboli E. et al. The EPIC Project: rationale and study design. European Prospective Investigation into Cancer and Nutrition. *Int J Epidemiol. Int J of Epidemiol* 1997;26(1):S6-14. **[PMID:12639222]** Riboli E. et al. European Prospective Investigation into Cancer and Nutrition (EPIC): study populations and data collection. *Public Health Nutr* 2002;5(6B):1113-24.
Health Professionals Follow-up Study	HPFS	20	86	Nested case-control, USA	Self-report through bi-annual questionnaires. Verified by medical records and pathology report	Cohort participants alive at time of case diagnosis without cancer, matched on date of birth, ethnicity, date and time of day of blood collection, and fasting status	**[PMID: 1678444]** Rimm E. et al. Prospective study of alcohol consumption and risk of coronary disease in men. *Lancet* 1991;338:464-8.
The Melbourne Collaborative Cohort Study	MCCS	59	246	Nested case-control, Australia	Incident cases ascertained through national cancer registries	Controls were unaffected cohort participants	**[PMID: 12484128]** Giles GG. et al. The Melbourne Collaborative Cohort Study. *IARC Sci Publ* 2002;156:69-70.

Supplementary Table 1. Description and study design of studies included in the discovery and replication

Study Name	Study Abbreviation	No. CLL Cases[a]	No. Controls[a]	Design, location	Source of cases	Source of controls	Study Reference
Nurses' Health Study	NHS	18	90	Nested case-control, USA	Self-report through bi-annual questionnaires. Verified by medical records and pathology report	Cohort participants alive at time of case diagnosis without cancer, matched on date of birth, ethnicity, date and time of day of blood collection, and fasting status	**[PMID: 15864280]** Colditz GA. et al. The Nurses' Health Study: lifestyle and health among women. *Nat Rev Cancer* 2005;5:388-96. **[PMID: 7658481]** Hankinson SE. et al. Alcohol, height and adiposity in relation to estrogen and prolactin levels in postmenopausal women. *J Natl Cancer Inst* 1995;87:1297-302.
New York University Women's Health Study	NYU-WHS	10	56	Nested case-control, USA	Self-report through questionnaires every 2-4 years, confirmed by medical and pathology records; and linkages to tumor registries of NY, NJ and Florida and NDI	Cohort participants selected by incidence density sampling (alive and free of cancer at time of case diagnosis)	**[PMID: 7707406]** Toniolo P. et al. A prospective study of endogenous estrogens and breast cancer in postmenopausal women. *J Natl Cancer Inst* 1995; 87:190-7. **[PMID: 20373009]** Gu Y. et al. Circulating cytokines and risk of B-cell non-Hodgkin lymphoma: a prospective study. *Cancer Causes Control* 2010; 21(8):1323-33.
Prostate, Lung, Colorectal, and Ovarian Cancer Screening Trial	PLCO	293	3076[b]	Nested case-control, USA	Self-report through annual questionnaires. Verified by medical records and pathology report	Cohort participants alive at time of case diagnosis without cancer diagnosis	**[PMID: 20494998]** Troy JD, et al. Associations between anthropometry, cigarette smoking, alcohol consumption, and non-Hodgkin lymphoma in the Prostate, Lung, Colorectal, and Ovarian Cancer Screening Trial. *Am J Epidemiol* 2010;171:1270-81. **[PMID: 16054167]** Hayes RB et al. Methods for etiologic and early marker investigations in the PLCO trial. *Mutat Res* 2005;592:147-54.
Women's Health Initiative	WHI	243	395	Nested case-control, USA	Self-report through semi-annual clinic visits or annual contact. Verified through medical records	Cohort participants without a diagnosis of cancer	**[PMID: 14575938]** Anderson GL, et al. Implementation of the Women's Health Initiative study design. *Ann Epidemiol* 2003 Oct;13(9 Suppl):S5-17.

Supplementary Table 1. Description and study design of studies included in the discovery and replication

Study Name	Study Abbreviation	No. CLL Cases[a]	No. Controls[a]	Design, location	Source of cases	Source of controls	Study Reference
British Columbia Non-Hodgkin Lymphoma Study	BC	28	390	Population-based case-control study, Canada	First primary NHL diagnosis from Vancouver and Victoria metropolitan areas identified through the BC Cancer Registry (excluding HIV-infected and post-transplant cases)	Controls from the same areas, matched on area, age, and sex ascertained from the British Columbia Health Insurance files	**[PMID:17722095]** Spinelli JJ. et al. Organochlorines and risk of non-Hodgkin lymphoma. *Int J Cancer* 2007; 121(12):2767-75.
Epidemiology & Genetics Unit Lymphoma Case-Control study	ELCCS		461	Population-based case-control study, UK	Cases were patients aged between 18-69 residing in predefined geographic areas and newly diagnosed with NHL between 1998 and 2003. Diagnoses were pathologically confirmed and coded to the WHO Classification for Oncology	For each case, one age- and sex- matched control was randomly selected from population based general practice registers	**[PMID: 15456990]** Willett EV. et al. Tobacco and alcohol consumption and the risk of non-Hodgkin lymphoma. *Cancer Causes Control* 2004;15:771-80. **[PMID: 19736055]** Worrillow L. et al. Polymorphisms in the nucleotide excision repair gene ERCC2/XPD and risk of non-Hodgkin lymphoma. *Cancer Epidemiol* 2009;33(3-4):257-60. **[PMID 20832384]** Crouch S. et al. Illness patterns prior to diagnosis of lymphoma: analysis of UK medical records. *Cancer Epidemiol* 2001;35(2):145-50.
Multicenter Italian study on gene-environment interactions in lymphoma etiology: translational aspects	Italian GxE	14	54	Population-based case-control study, Italy	First primary NHL diagnosis identified in the Hematology Departments of the participating centers	Controls are randomly selected among residents in the study areas or among patients admitted for selected diagnosis in the referring hospital of the same areas of the respective cases, frequency matched to cases by gender, age, and residence	
National Cancer Institute-Surveillance, Epidemiology, and End Results Interdisciplinary Case-Control Study of Non-Hodgkin's Lymphoma	NCI-SEER	91	689	Population-based case-control study, USA	First primary NHL diagnosis identified through 4 SEER registries (excluding HIV-infected cases)	Controls from the same areas, matched on area, age, and race ascertained though random digit dialing (<64 years of age) and CMMS files (≥65 years of age)	**[PMID: 15342441]** Chatterjee N. et al. Risk of non-Hodgkin's lymphoma and family history of lymphatic, hematology, and other cancers. *CEBP* 2004;13:1415-21. **[PMID: 17018637]** Wang SS. et al. Common genetic variants in proinflammatory and other immunoregulatory genes and risk for non-Hodgkin lymphoma. *Cancer Res* 2006;66(19):9771-80.

Supplementary Table 1. Description and study design of studies included in the discovery and replication

Study Name	Study Abbreviation	No. CLL Cases[a]	No. Controls[a]	Design, location	Source of cases	Source of controls	Study Reference
NSW non-Hodgkin lymphoma study	NSW	15	397	Population-based case-control study, Australia	Incident NHL diagnosis identified through New South Wales (NSW) or Australian Capital Territory (ACT) cancer registry (excluding HIV-infected cases and transplant recipients)	Controls randomly selected from electoral rolls, matched on age, sex and State of residence at diagnosis	**[PMID: 15095310]** Hughes AM, et al. Pigmentary characteristics, sun sensitivity and non-Hodgkin lymphoma. *IJC* 2004;110:429-34.
Scandinavian Lymphoma Etiology Study	SCALE	402	301	Population-based case-control study, Scandinavia	Patients with incident primary NHL diagnosed through rapid case-ascertainment network in Sweden and Denmark	Frequency matched (age in 10 year intervals, sex and country) population controls prospectively identified every 6 months in nationwide population registers (incidence density sampling).	**[PMID: 15687363]** Smedby KE. et al. Ultraviolet radiation exposure and risk of malignant lymphomas. *J Natl Cancer Inst* 2005;97(3)199-209.
Molecular Epidemiology of non-Hodgkin lymphoma	UCSF	23	10	Population-based case-control study, USA	RCA/SEER Incident NHL diagnosis for patients diagnosed in hospitals in 6 San Francisco Bay Area Counties and who were residents of the Bay Area at the time of diagnosis	Controls ascertained through RDD were frequency matched to cases on age in 5-year groups, sex and county of residence; Random sampling of CMS lists for person residing in the same 6 Bay Area counties were used to supplement recruitment of controls aged 65+	[**PMID: 1863612**] Skibola CF. et al. Polymorphisms in the estrogen receptor 1 and vitamin C and matrix metalloproteinase gene families are associated with susceptibility to lymphoma. *PLoS One* 2008; 30;3(7):e2815.
Population-based case-control study in Connecticut women	Yale	41	504	Population-based case-control study, USA	First primary NHL diagnosis identified through the Rapid Case Shared Resources from all the hospitals in Connecticut	Population-based controls through random digit dialing for cases <65 years and Medicare files for ≥65 years	**[PMID: 19822571]** Zhang Y et al. Genetic variations in xenobiotic metabolic pathway genes, personal hair dye use and risk of non-Hodgkin lymphoma. *Am J Epidemiol* 2009;170(10):1222-30.
Environmental and genetic risks factors study in adult lymphoma	ENGELA	51	278	Hospital-based case-control study, France	Recent diagnosis of a NHL as per the WHO classification (ICD-O-3) / Cases with AIDS or on immunosuppressant drugs were not eligible. Path reports for 100%, slides review for selected NHL	Hospitalized in the same hospitals as the cases, for any reason except cancer, an accident or a disease directly related to the subject's occupation, smoking, or alcohol consumption. HIV negative.	**[PMID: 18781390]** Monnereau A. et al. Cigarette smoking, alcohol drinking, and risk of lymphoid neoplasms: results of a French case-control study. *Cancer Causes Control* 2008;19(10):1147-60.

Supplementary Table 1. Description and study design of studies included in the discovery and replication

Study Name	Study Abbreviation	No. CLL Cases[a]	No. Controls[a]	Design, location	Source of cases	Source of controls	Study Reference
Epilymph case-control study in six European countries	EpiLymph	211	1172	Multicenter case-control study, hospital-based and population-based, Europe	First primary lymphoma diagnosis (according to the 2001 WHO classification of lymphoma)	Controls from Germany and Italy were randomly selected by sampling from the general population, matched to cases on gender, age, and residence area. The rest of the centers used matched hospital controls, with eligibility criteria limited to diagnoses other than cancer, infectious or immune-related diseases.	**[PMID:16557575]** Besson H. et al. Tobacco smoking, alcohol drinking and non-Hodgkin's lymphoma: A European multicenter case-control study (Epilymph). *Int J Cancer* 2006;119:901-8.
Iowa-Mayo SPORE Molecular Epidemiology Resource	Iowa-Mayo SPORE	249		Clinic-based case registry, USA	Consecutive patients with newly diagnosed, histologically-confirmed non-Hodgkin lymphoma (excluding HIV-infected cases) who were residents of US	N/A	**[PMID: 20713849]** Drake MT. et al. Vitamin D insufficiency and prognosis in non-Hodgkin's lymphoma. *J Clin Oncol* 2010;28:4191-8.
Mayo Clinic Case-Control Study of NHL and CLL	Mayo Case-Control	139	911	Clinic-based case-control study, USA	Consecutive patients with newly diagnosed, histologically-confirmed non-Hodgkin lymphoma (excluding HIV-infected cases) who were residents of Minnesota, Iowa or Wisconsin	Controls were selected from patients seen in the general medicine clinics at Mayo with a pre-scheduled general medical examination, frequency on age, sex, and geographic region	**[PMID: 2168612]** Cerhan JR. et al. Design and validity of a clinic-based case-control study on the molecular epidemiology of lymphoma. *Int J Mol Epidemiol Genet* 2011;2(2):95-113.
Memorial-Sloan Kettering Lymphoproliferative disorders Study	MSKCC	37	9	Hospital-based case-study and NYCP controls, USA	Hospital clinic based ascertainment in a tertiary referral center	NYCP controls from same geographic area	**[PMID: 23349640]** Vijai J. et al. Susceptibility loci associated with specific and shared subtypes of lymphoid malignancies. *PLoS Genet* 2013;9(1):e1003220.
**DISCOVERY – ADDITIONAL GWAS**							
Utah Chronic Lymphocytic Leukemia Study	UTAH	355	420	Mixed: clinic- and population-based cases and controls, USA	Prevalent cases from Huntsman Cancer Hospital's Hematology Clinics and Prevalent cases identified in the Utah Cancer Registry, verified by medical records and pathology report	Controls from the same area frequency matched by sex and birth cohort using the Utah Population Database	

Supplementary Table 1. Description and study design of studies included in the discovery and replication

Study Name	Study Abbreviation	No. CLL Cases[a]	No. Controls[a]	Design, location	Source of cases	Source of controls	Study Reference
Genetic Epidemiology of CLL (GEC) Consortium	GEC	391	296	Family Study, USA	Eligible case probands with verified history of CLL in families were identified by investigators at Duke University, Mayo Clinic, the University of Texas M. D. Anderson Cancer Center, the National Cancer Institute (NCI), the University of Minnesota/Minneapolis Veterans Administration Medical Center, the University of California-San Diego, and the University of Utah	N/A	**[PMID: 21131588]** Slager SL, et al. Genome-wide association study identifies a novel susceptibility locus at 6p21.3 among familial CLL. *Blood* 2011;117:1911-16.
Molecular Epidemiology of non-Hodgkin lymphoma	UCSF	214	753	Population-based case-control study, USA	RCA/SEER Incident NHL diagnosis for patients diagnosed in hospitals in 6 San Francisco Bay Area Counties and who were residents of the Bay Area at the time of diagnosis	Controls ascertained through RDD were frequency matched to cases on age in 5-year groups, sex and county of residence; Random sampling of CMS lists for person residing in the same 6 Bay Area counties were used to supplement recruitment of controls aged 65+	[**PMID: 19620980**] Conde L. et al. Genome-wide association study of follicular lymphoma identifies a risk locus at 6p21.32. *Nat Genet* 2010;42(8):661-4. [**PMID: 22697504**] Mikhak B. et al. Intake of vitamins d and a and calcium and risk of non-Hodgkin lymphoma: San Francisco Bay Area population-based case-control study. *Nutr Cancer* 2012;64(5):674-84.
**REPLICATION STUDIES**							
Genetic Epidemiology of CLL (GEC) Consortium[d]	GEC	136	0[c]	Family Study, USA	Eligible case probands with verified history of CLL in families were identified by investigators at Duke University, Mayo Clinic, the University of Texas M. D. Anderson Cancer Center, the National Cancer Institute (NCI), the University of Minnesota/Minneapolis Veterans Administration Medical Center, the University of California-San Diego, and the University of Utah	N/A	**[PMID: 21131588]** Slager SL, et al. Genome-wide association study identifies a novel susceptibility locus at 6p21.3 among familial CLL. Blood. Blood 2011;117:1911-16.

Supplementary Table 1. Description and study design of studies included in the discovery and replication

Study Name	Study Abbreviation	No. CLL Cases[a]	No. Controls[a]	Design, location	Source of cases	Source of controls	Study Reference
Iowa-Mayo SPORE Molecular Epidemiology Resource[d]	Iowa-Mayo SPORE	123	0[c]	Clinic-based case registry, USA	Consecutive patients with newly diagnosed, histologically-confirmed non-Hodgkin lymphoma (excluding HIV-infected cases) who were residents of US	N/A	**[PMCID:PMC2953973]** Drake MT. et al. Vitamin D insufficiency and prognosis in non-Hodgkin's lymphoma. *J Clin Oncol* 2010;28:4191-8.
Mayo Clinic Case-Control Study of NHL[d]	Mayo Case-Control	247	518	Clinic-based case-control study, USA	Consecutive patients with newly diagnosed, histologically-confirmed non-Hodgkin lymphoma (excluding HIV-infected cases) who were residents of Minnesota, Iowa or Wisconsin	Controls were selected from patients seen in the general medicine clinics at Mayo with a pre-scheduled general medical examination, frequency on age, sex, and geographic region	**[PMCID:PMC3110384]** Cerhan JR. et al. Design and validity of a clinic-based case-control study on the molecular epidemiology of lymphoma. *Int J Mol Epidemiol Genet* 2011;2(2):95-113.
Multicase-control study in Spain[d]	MCC-Spain	567	1,912	Case-control, Spain	Identified through medical records and/or cytogenetics laboratories in the hospital participating in the study	Participants without personal history of lymphoprolyferative disorder from the general population and frequency matched to CLL by age, sex and area of residence	**[PMID: 25613680]** Population-based multicase-control study in common tumors in Spain (MCC-Spain): rationale and study design. *Gac Sanit*. 2015 Jul-Aug;29(4):308-15
MD Anderson lymphoma case control study[d]	MDALyms	644	644	Case-control, Texas	MD Anderson Cancer Center	Kelsey Seybold Clinics	
Memorial-Sloan Kettering Lymphoproliferative disorders Study[d]	MSKCC	300	379	Hospital-based case-study and NYCP controls, USA	Hospital clinic based ascertainment in a tertiary referral center	NYCP controls from same geographic area	**[PMID: 23349640]** Vijai J. et al. Susceptibility loci associated with specific and shared subtypes of lymphoid malignancies. *PLoS Genet* 2013;9(1):e1003220.
NCI Replication Study	NCI Rep	173	4296	Mixed study of population and hospital-based cases and controls	CLL cases from the stage 1 studies that did not have sufficient DNA for scanning or failed in scanning due to low completion.	Controls from the stage 1 studies that were not scanned or failed scanning due to low completion.	

Supplementary Table 1. Description and study design of studies included in the discovery and replication

Study Name	Study Abbreviation	No. CLL Cases[a]	No. Controls[a]	Design, location	Source of cases	Source of controls	Study Reference
Utah/Sheffield Chronic Lymphocytic Leukemia Collaborative Study	UTAH-SHEFFIELD	236	227	Mixed: clinic- and population-based cases and controls	UTAH: Prevalent cases from Huntsman Cancer Hospital's Hematology Clinics and Prevalent cases identified in the Utah Cancer Registry, verified by medical records and pathology report. SHEFFIELD: Prevalent cases from National Health Service Hospitals in the North Trent region of the UK	Controls ascertained from the Utah Population Database	

[a]Number of cases and controls with DNA available.
[b]Controls scanned previously on the Illumina Omni2.5
[c]Controls from the Mayo Case-Control study were used for analysis.
[d]Not included in previous manuscript by Berndt et al. (Nature Genetics, 2013)

Supplementary Table 2. Information on genotyping methods, quality control, imputation, and analysis for GWAS included in the discovery meta-analysis

Study	Sample QC				Genotyping and Imputation							
		Inclusion/exclusion criteria					Inclusion criteria for Imputation					
	No. of cases /controls in file	Minimum sample call rate for inclusion	Exclusions	No.cases /controls after exclusions	Platform	Genotype calling algorithm	MAF	SNP Call rate*	P for HWE	SNPs that met QC criteria	Imputation Software	SNPs in meta-analysis[a]
**Discovery**												
NCI	2301/6390[a]	>=93%	1) Abnormal heterozygosity; 2) gender discordance; 3) unexpected duplicates; 4) Non CEU	2179/6221	Illumina OmniExpress/ Omni2.5	BeadStudio (GenCall)	>= 0.01	>=0.95	>=1e-6	608811	IMPUTE2	8478083
GEC	391/296	>=95%	1) Non CEU ; 2) PCA outliers	387/294	Affymetrix 6.0	Birdseed	>= 0.01	>=0.95	>=1e-6	687578	IMPUTE2	8499108
Utah	331/420	>=95%	1) Abnormal heterozygosity; 2) Non CEU; 3) Incomplete phenotype	321/405	Illumina HumanHap 610K	BeadStudio (GenCall)	>= 0.01	>=0.95	>=1e-6	512171	IMPUTE2	8476283
UCSF2	214/751	>=95%	1) Abnormal heterozygosity; 2) PCA outlier	213/747	Illumina HumanCNV370-Duo	BeadStudio (GenCall)	>= 0.01	>=0.95	>=1e-6	290523	IMPUTE2	8518031

[a]For all studies, SNPs with MAF>0.01 or INFO>0.3 were filtered out prior to analysis.

Supplementary Table 3. Characteristics of the subjects included in the discovery and replication

Study	No. of Subjects		% Male		Mean (SD) Age	
	Case	Control	Case	Control	Case	Control
**Discovery GWAS**						
NCI	2179	6221	55.70%	72.80%	65 (9.3)	66 (10.1)
GEC	387	294	65.10%	63.30%	61 (11.1)	63 (11.2)
UCSF2	213	747	63.80%	57.70%	64 (11.2)	61 (13.0)
Utah	321	405	59.80%	56.00%	63 (10.3)	64 (10.7)
**Total**	**3,100**	**7,667**	**57.87%**	**70.05%**	**64(9.9)**	**65(10.6)**
**Replication studies**						
Mayo/IA-Mayo SPORE/GEC	506	518	62.8%	60.0%	62.2 (10.7)	62.6 (11.7)
MCC-Spain	143	140	51.2%	48.8%	67.0 (11.0)	67.0 (10.0)
MD Anderson	644	644	64.8%	64.8%	58.0 (10.2)	60.9 (10.0)
MSKCC	300	379	65.0%	17.9%	60.5 (11.5)	57.7 (11.7)
NCI Rep	131	3624	68.7%	44.3%	65.5 (9.17)	60.8 (14.4)
UTAH-SHEFFIELD	234	225	64.1%	39.6%	65.1 (10.4)	67.3 (10.8)
**Total**	**1,958**	**5,530**	**63.5%**	**46.3%**	**61.5**	**61.2**

Supplementary Table 4. Published CLL SNP and most significant SNP within 1 Mb of the published SNP: results from the discovery meta-analysis

Locus	Nearest gene	Position	Published SNP	Lead SNP in meta-analysis	r^2 with published SNP	Effect allele/ Other allele	No. cases	No. controls	EAF	OR	CI	P	P_{het}	I^2	Reference for published SNP
2p22.2	*QPCT*	37596089	rs3770745	rs3770745		T/C	3096	7663	0.229	1.22	(1.14-1.32)	1.36E-07	0.05	61.0	Berndt et al.
2q13	*ACOXL*	111797458	rs17483466			G/A	3100	7667	0.194	1.39	(1.29-1.50)	**5.96E-18**	0.87	0	Di Bernardo et al.
	ACOXL	111831793		rs58055674	0.629	C/T	3097	7665	0.173	1.44	(1.33-1.56)	**5.23E-20**	0.40	0	
2q33.1	*CASP8*	202111380	rs3769825			G/A	3100	7667	0.545	0.85	(0.80-0.90)	2.08E-07	0.96	0	Berndt et al.
	FAM126B	201909515		rs13015798	0.160	G/A	3096	7663	0.327	0.83	(0.77-0.88)	**2.70E-08**	0.60	0	
2q37.1	*SP140*	231091223	rs13397985			G/T	3100	7667	0.182	1.47	(1.36-1.59)	**6.17E-23**	0.91	0	Di Bernardo et al.
	SP140	231098071		rs7557418	1	A/C	3097	7666	0.181	1.47	(1.36-1.59)	**4.93E-23**	0.96	0	
2q37.3	*FARP2*	242371101	rs757978			T/C	3100	7667	0.097	1.29	(1.17-1.42)	3.15E-07	0.64	0	Crowther-Swanepoel et al.
	FARP2	242294913		rs3755397	0.866	G/A	3098	7663	0.100	1.36	(1.23-1.50)	**8.25E-10**	0.48	0	
3q26.2	*MYNN*	169492101	rs10936599			T/C	3100	7667	0.249	0.86	(0.80-0.93)	5.01E-05	0.77	0	Speedy et al.
	MYNN	169497585		rs1317082	1	G/A	3100	7667	0.249	0.86	(0.80-0.92)	3.73E-05	0.75	0	
4q25	*LEF1*	109016824	rs898518			A/C	3100	7666	0.585	1.17	(1.10-1.25)	4.57E-07	0.68	0	Berndt et al.
	LEF1	109026414		rs2003869	0.950	G/A	3096	7663	0.567	1.19	(1.11-1.26)	1.42E-07	0.70	0	
4q26	*CAMK2D*	114683844	rs6858698			C/G	3096	7663	0.172	0.96	(0.87-1.05)	0.32	0.23	30	Speedy et al.
	ANK2	114000767		rs79210227	0.001	C/A	3096	7664	0.021	1.57	(1.25-1.98)	9.98E-05	0.62	0	
5p15.33	*TERT*	1279790	rs10069690			T/C	3097	7664	0.250	1.21	(1.12-1.30)	5.56E-07	0.59	0	Berndt et al.; Speedy et al.
	TERT	1284653		rs139996880	0.378	A/G	3096	7663	0.165	1.29	(1.18-1.42)	6.08E-08	0.71	0	
6p25.3	*IRF4*	411064	rs872071			G/A	3099	7666	0.494	1.35	(1.27-1.43)	**1.32E-21**	0.42	0	Di Bernardo et al.
	IRF4	409119		rs9391997	1	G/A	3097	7665	0.494	1.35	(1.27-1.43)	**8.76E-22**	0.42	0	
6p21.32	*HLA-DRB1, HLA-DRB5*	32578082	rs674313			T/C	3098	7665	0.245	1.14	(1.06-1.22)	0.0003	0.52	0	Slager et al.
	HLA-DQB1	32568346		rs9270750	0.021	A/G	3096	7663	0.543	1.32	(1.23-1.41)	**5.52E-16**	0.06	59	
6p21.31	*BAK1*	33546837	rs210142			C/T	3099	7666	0.710	1.20	(1.12-1.29)	1.59E-07	0.10	52	Slager et al.
	BAK1	33546930		rs210143	1	C/T	3099	7666	0.706	1.21	(1.13-1.30)	9.52E-08	0.07	58	
6q25.2	*IPCEF1, OPRM1*	154478440	rs2236256			A/C	3099	7666	0.555	0.95	(0.90-1.01)	0.12	0.99	0	Speedy et al.
	IPCEF1	154583379		rs7761411	0.289	C/T	3098	7665	0.511	0.87	(0.82-0.93)	1.45E-05	0.88	0	
7q31.33	*POT1*	124462661	rs17246404			T/C	3100	7667	0.283	0.90	(0.84-0.96)	0.002	0.39	0.9	Speedy et al.
	LOC101928211	123959169		rs73233504	0.009	A/G	3096	7664	0.061	1.43	(1.22-1.68)	1.13E-05	0.45	0	
8q22.3	*ODF1*	103578874	rs2511714	rs2511714	1	G/T	3096	7663	0.391	1.19	(1.11-1.27)	2.16E-07	0.09	54	Berndt et al.; Speedy et al.
8q24.21	*CASC19*	128192981	rs2456449			G/A	3098	7666	0.340	1.25	(1.17-1.33)	**1.98E-11**	0.83	0	Crowther-Swanepoel et al.
	CASC19	128195334		rs140099016	0.872	C/T	3096	7663	0.294	1.28	(1.19-1.37)	**2.23E-12**	0.75	0	
9p21.3	*CDKN2B-AS1*	22206987	rs1679013			T/C	3097	7664	0.473	0.84	(0.79-0.89)	**1.84E-08**	0.27	24	Berndt et al.

Supplementary Table 4. Published CLL SNP and most significant SNP within 1 Mb of the published SNP: results from the discovery meta-analysis

Locus	Nearest gene	Position	Published SNP	Lead SNP in meta-analysis	r^2 with published SNP	Effect allele/ Other allele	No. cases	No. controls	EAF	OR	CI	P	P_{het}	I^2	Reference for published SNP
	DMRTA1	22336996		rs1359742	0.510	C/G	3098	7664	0.501	0.83	(0.78-0.89)	**6.68E-09**	0.78	0	
10q23.31	*ACTA2,FAS*	90759724	rs4406737	rs4406737		G/A	3099	7666	0.570	1.27	(1.19-1.35)	**9.06E-14**	0.04	63	Berndt et al.
11p15.5	*C11orf21*	2311152	rs7944004			G/T	3099	7666	0.509	0.84	(0.79-0.90)	8.61E-08	0.76	0	Berndt et al.
	C11orf21	2321095		rs2521269	0.455	A/C	3096	7663	0.542	0.84	(0.79-0.89)	**3.52E-08**	0.86	0	
11q24.1	*GRAMD1B*	123361397	rs735665			A/G	3100	7667	0.196	1.65	(1.53-1.77)	**1.10E-39**	0.29	19	Di Bernardo et al.
	GRAMD1B	123355391		rs35923643	1	G/A	3097	7663	0.196	1.66	(1.54-1.79)	**2.05E-40**	0.29	20	
12q24.13	*OAS3*	113380008	rs10735079			A/G	3099	7665	0.649	0.90	(0.84-0.96)	0.0009	0.35	8.7	Sava et al.
	RPH3A	113159848		rs66517857	0.008	T/A	3097	7664	0.165	1.17	(1.08-1.27)	0.0002	0.78	0	
15q15.1	*BMF*	40403657	rs8024033			G/C	3096	7663	0.486	0.82	(0.77-0.88)	**2.71E-10**	0.03	67	Berndt et al.
	BMF	40397936		rs539846	0.932	T/G	3096	7663	0.507	1.22	(1.15-1.30)	**6.62E-10**	0.04	65	
15q21.3	*RFX7*	56340896	rs7169431			G/A	3099	7666	0.917	0.79	(0.71-0.88)	1.21E-05	0.23	30	Crowther-Swanepoel et al.
	MNS1	56780767		rs72742684	0.202	T/C	3096	7663	0.108	1.43	(1.30-1.58)	**1.63E-13**	0.05	62	
15q23	*PCAT29*	70018990	rs7176508			G/A	3099	7667	0.616	0.76	(0.72-0.81)	**8.36E-18**	0.72	0	Di Bernardo et al.
	PCAT29	69989505		rs2052702	0.927	C/A	3096	7663	0.614	0.76	(0.71-0.81)	**4.39E-18**	0.65	0	
15q25.2	*CPEB1*	83254708	rs783540			G/A	3099	7667	0.385	1.07	(1.01-1.14)	0.03	0.55	0	Crowther-Swanepoel et al.
	BNC1	83970546		rs7172622	0.044	G/A	3096	7663	0.58	0.90	(0.84-0.96)	0.001	0.93	0	
16q24.1	*IRF8*	85975659	rs305061			T/C	3100	7667	0.656	1.16	(1.08-1.23)	1.18E-05	0.32	14	Crowther-Swanepoel et al.
	IRF8	85928621		rs391855	5.1E-06	T/A	3096	7663	0.434	0.73	(0.69-0.78)	**1.06E-22**	0.37	3.9	
18q21.32	*PMAIP1*	57622287	rs4368253	rs4368253		C/T	3099	7665	0.677	1.18	(1.11-1.26)	7.90E-07	0.42	0	Berndt et al.
18q21.33	*BCL2*	60793549	rs4987855			T/C	3099	7666	0.094	0.69	(0.62-0.77)	**6.74E-11**	0.57	0	Berndt et al.
	BCL2	60793494		rs4987856	1	T/C	3098	7666	0.094	0.69	(0.62-0.77)	**3.55E-11**	0.58	0	
19q13.3	*PRKD2*	47207654	rs11083846			A/G	3098	7665	0.232	1.10	(1.02-1.18)	0.01	0.18	38	Di Bernardo et al.
	SAE1	47696336		rs10423223	0.028	G/A	3098	7664	0.253	1.14	(1.06-1.22)	0.0002	0.23	31	

Supplementary Table 5. Meta-analysis results for previously published loci for CLL

							Reported Results from Original Publication				Meta-analysis of 4 CLL GWAS (NCI, GEC, Utah, UCSF2)			Meta-analysis of 4 CLL GWAS + UK-CLL-1+UK-CLL-2[a]		
Locus	Nearest gene	Position	SNP[b]	r^2[c]	Effect allele	Other allele	No cases/ No. controls	OR	P	Reference	No cases/ No. controls	OR	P	No cases/ No. controls	OR	P
2p22.2	*QPCT*	37596089	rs3770745*	1	T	C	3097/7663	1.24	**1.68E-08**	Berndt et al.	3096/7663	1.22	1.36E-07			
	QPCT	37559355	rs6734118	0.36	A	C	1739/5199	1.17	0.001	Speedy et al.	3100/7667	1.15	2.55E-04	4839/12866	1.15	1.17E-06
2q13	*ACOXL*	111797458	rs17483466*	1	G	A	1524/3094	1.39	**2.36E-10**	Di Bernardo et al.	3100/7667	1.39	**5.96E-18**	4837/12865	1.39	**6.93E-28**
	ACOXL	111616104	rs13401811*	0.04	A	G	3839/12264	0.71	**2.08E-18**	Berndt et al.	3097/7666	0.71	**4.08E-17**			
	ACOXL	111600519	rs13395354	0.04/0.97	T	C	1739/5199	0.75	**2.24E-08**	Speedy et al.	3100/7666	0.71	**1.86E-16**	4839/12865	0.73	**3.24E-23**
2q33.1	*CASP8*	202111380	rs3769825*	1	G	A	3885/12471	0.84	**2.50E-09**	Berndt et al.	3100/7667	0.85	2.08E-07	4837/12865	0.87	**1.03E-08**
2q37.1	*SP140*	231091223	rs13397985*	1	G	T	1500/3053	1.41	**5.40E-10**	Di Bernardo et al.	3100/7667	1.47	**6.17E-23**	4837/12865	1.46	**2.32E-34**
2q37.3	*FARP2*	242371101	rs757978*	1	T	C	2489/5770	1.39	**2.11E-09**	Crowther-Swanepoel et al.	3100/7667	1.29	3.15E-07	4837/12865	1.27	**5.33E-10**
3q26.2	*MYNN*	169492101	rs10936599*	1	T	C	2868/8329	0.79	**1.74E-09**	Speedy et al.	3100/7667	0.86	5.01E-05	5627/15626*	0.83	**1.04E-11**
4q25	*LEF1*	109016824	rs898518*	1	A	C	3879/12441	1.20	**4.24E-10**	Berndt et al.	3100/7666	1.17	4.57E-07	4837/12864	1.16	**1.09E-08**
4q26	*CAMK2D*	114683844	rs6858698	1	C	G	2823/8316	1.31	**3.07E-09**	Speedy et al.	3096/7663	0.96	0.32	5597/15609*	1.11	**0.002**
5p15.33	*TERT*	1279790	rs10069690*	1	T	C	5206/17296	1.20	**1.12E-10**	Speedy et al.	3097/7664	1.21	5.56E-07	4834/12862	1.20	**2.54E-10**
	CLPTM1L	1344458	rs31490	0.002	A	G	2880/8277	1.18	1.72E-07	Speedy et al.	3098/7665	1.03	0.43	5637/15571*	1.10	1.68E-05
6p25.3	*IRF4*	411064	rs872071*	1	G	A	1517/3102	1.54	**1.91E-20**	Di Bernardo et al.	3099/7666	1.35	1.32E-21	4836/12864	1.36	**3.50E-36**
6p21.32	*HLA-DRB1*	32578082	rs674313	1	T	C	198/794	1.87	1.98E-07	Slager et al.	3098/7665	1.14	3.04E-04			
	HLA-DQB1	32626272	rs9273363*	0.06	A	C	3097/7664	1.26	**3.66E-11**	Berndt et al.	3097/7664	1.26	**3.66E-11**			
	HLA-DQA1	32611641	rs9273012	0.49/0.03	G	A	1739/5199	1.18	4.34E-04	Speedy et al.	3098/7664	1.18	3.11E-06	4837/12863	1.18	**5.21E-09**
6p21.31	*BAK1*	33546837	rs210142*	1	C	T	1982/5778	1.40	**9.47E-16**	Slager et al.	3099/7666	1.20	1.59E-07			
	BAK1	33540209	rs210134	0.97	G	A	1982/5778	1.37	**1.03E-12**	Slager et al.	3099/7667	1.19	1.02E-06	4836/12865	1.23	**8.68E-15**
6q25.2	*IPCEF1*	154478440	rs2236256*	1	A	C	2828/8262	0.81	**1.50E-10**	Speedy et al.	3099/7666	0.95	0.12	5590/15570*	0.88	**1.91E-08**
7q31.33	*POT1*	124462661	rs17246404*	1	T	C	2846/8298	0.82	**3.40E-08**	Speedy et al.	3100/7667	0.90	0.002	5608/15597*	0.86	**6.38E-09**
8q22.3	*ODF1*	103578874	rs2511714*	1	G	T	5231/13174	1.16	**2.90E-09**	Speedy et al.	3096/7663	1.19	2.16E-07	4833/12861	1.16	**1.47E-08**
8q24.21	*CASC19*	128192981	rs2456449*	1	G	A	2441/5655	1.26	**7.84E-10**	Crowther-Swanepoel et al.	3098/7666	1.25	**1.98E-11**			
	CASC19	128188019	rs2466024	0.71	A	G	1739/5199	1.21	3.24E-06	Speedy et al.	3099/7665	1.19	5.36E-08	4836/12863	1.20	**8.70E-13**
9p21.3	*CDKN2B-AS1*	22206987	rs1679013*	1	T	C	3482/12148	0.84	**1.27E-08**	Berndt et al.	3097/7664	0.84	**1.84E-08**			
	DMRTA1	22336954	rs1359741	0.55	A	G	1739/5199	0.91	0.02	Speedy et al.	3100/7666	0.86	7.15E-07	4839/12865	0.88	9.95E-08
10q23.31	*ACTA2,FAS*	90759724	rs4406737*	1	G	A	3481/12170	1.27	**1.22E-14**	Berndt et al.	3099/7666	1.27	**9.06E-14**	4836/12864	1.25	**3.26E-19**
11p15.5	*C11orf21*	2311152	rs7944004*	1	G	T	3869/12476	0.83	**2.15E-10**	Berndt et al.	3099/7666	0.84	8.61E-08	4836/12864	0.86	**2.00E-09**
11q24.1	*GRAMD1B*	123361397	rs735665*	1	A	G	1504/3101	1.45	**3.78E-12**	Di Bernardo et al.	3100/7667	1.65	**1.10E-39**	4837/12865	1.64	**4.94E-62**
12q24.13	*OAS3*	113380008	rs10735079*	1	A	G	2553/6006	0.85	**2.34E-08**	Sava et al.	3099/7665	0.90	9.00E-04	4838/12864	1.15	6.30E-08
15q15.1	*BMF*	40403657	rs8024033*	1	G	C	3096/7663	0.82	**2.71E-10**	Berndt et al.	3096/7663	0.82	**2.71E-10**			
	BMF	40387971	rs11637681	0.30	G	A	1739/5199	0.83	1.06E-04	Speedy et al.	3099/7666	0.88	6.38E-04	4838/12865	0.86	4.25E-07

Supplementary Table 5. Meta-analysis results for previously published loci for CLL

							Reported Results from Original Publication				Meta-analysis of 4 CLL GWAS (NCI, GEC, Utah, UCSF2)			Meta-analysis of 4 CLL GWAS + UK-CLL-1+UK-CLL-2[a]		
Locus	Nearest gene	Position	SNP[b]	r^{2c}	Effect allele	Other allele	No cases/ No. controls	OR	P	Reference	No cases/ No. controls	OR	P	No cases/ No. controls	OR	P
15q21.3	*RFX7*	56340896	rs7169431	1	G	A	2461/5738	0.74	4.74E-07	Crowther-Swanepoel et al.	3099/7666	0.79	1.2E-05	4836/12864	0.76	**1.27E-10**
	MNS1	56775597	rs11636802*	0.20	G	A	3097/7666	1.41	**6.47E-13**	Berndt et al.	3097/7666	1.41	**6.47E-13**			
15q23	*PCAT29*	70018990	rs7176508*	1	G	A	1480/2991	0.73	**4.54E-12**	Di Bernardo et al.	3099/7667	0.76	**8.36E-18**	4836/12865	0.74	**1.61E-33**
15q25.2	*CPEB1*	83254708	rs783540	1	G	A	3931/7709	1.17	1.10E-07	Crowther-Swanepoel et al.	3099/7667	1.07	0.03			
	LOC283693	83318202	rs11631963	1	C	T	1739/5199	0.88	0.001	Speedy et al.	3099/7666	0.94	0.04	4838/12865	0.91	**0.0003**
16q24.1	*IRF8*	85975659	rs305061*	1	T	C	2470/5766	1.22	3.60E-07	Crowther-Swanepoel et al.	3100/7667	1.16	1.18E-05	4837/12865	1.17	**1.92E-09**
	IRF8	85944439	rs391525*	0.0001	G	A	503/794	0.55	**6.94E-11**	Slager et al.	3099/7666	0.76	**2.09E-16**			
	IRF8	85944823	rs2292982	0.0001	G	T	503/794	0.56	**2.13E-10**	Slager et al.	3099/7665	0.76	**3.03E-16**			
18q21.32	*PMAIP1*	57622287	rs4368253*	1	C	T	3882/12473	1.19	**2.51E-08**	Berndt et al.	3099/7665	1.18	7.90E-07			
	PMAIP1	57628926	rs7231647	0.75	A	G	1739/5199	1.18	4.41E-05	Speedy et al.	3100/7667	1.14	2.54E-05	4839/12866	1.16	**5.59E-09**
18q21.33	*BCL2*	60793549	rs4987855*	1	T	C	3883/12446	0.68	**2.66E-12**	Berndt et al.	3099/7666	0.69	**6.74E-11**	4836/12864	0.74	**3.51E-12**
	BCL2	60793921	rs4987852*	0.008	C	T	3880/12497	1.41	**7.76E-11**	Berndt et al.	3100/7667	1.43	**1.61E-09**	4837/12865	1.40	**7.00E-13**
19q13.3	*PRKD2*	47207654	rs11083846*	1	A	G	1518/3092	1.35	**3.96E-09**	Di Bernardo et al.	3098/7665	1.10	0.01			
	STRN4	47242992	rs4802322	0.91	A	G	1739/5199	1.22	1.48E-05	Speedy et al.	3098/7665	1.09	0.01	4837/12864	1.14	4.11E-06

[a]For the novel loci reported in Speedy et al., the replication results from the UK replication reported in Speedy et al. were also included in the meta-analysis. The Swedish replication reported in this paper was not included as many of the subjects overlap with subjects in our discovery meta-analysis.

[b]The SNPs with an asterisk are the previously reported loci included in the polygenetic score and pathway analyses. For loci containing more than one independent SNP, both (or all three for 2q13) independent SNPs were included in the polygenetic score analysis. For the pathway analyses, only one SNP per locus was chosen.

[c]R^2 with the first SNP listed for the locus.

Supplementary Table 6. Individual study results for SNPs taken forward for replication

SNP	Study	Genotyped or imputed (info score)[a]	No. of cases	No. of controls	Effect allele/ Other allele	EAF[b]	OR	CI	P	P_{het}	I^2
rs9880772	NCI GWAS	i (0.991)	2179	6221	A/G	0.466	1.17	(1.09-1.25)	2.00E-05		
rs9880772	Utah GWAS	i (0.989)	320	404	A/G	0.465	1.02	(0.83-1.26)	0.82		
rs9880772	GEC GWAS	i (0.936)	386	293	A/G	0.491	1.14	(0.91-1.43)	0.26		
rs9880772	UCSF2 GWAS	i (0.931)	212	746	A/G	0.435	1.41	(1.13-1.77)	0.002		
rs9880772	Mayo/ IA-M/GEC	g	502	516	A/G	0.458	1.25	(1.05-1.50)	0.01		
rs9880772	MD Anderson	g	640	637	A/G	0.455	1.20	(1.03-1.40)	0.02		
rs9880772	MSKCC	g	300	377	A/G	0.454	1.44	(0.54-0.89)	0.004		
rs9880772	Utah-Sheffield	g	228	225	A/G	0.476	1.14	(0.86-1.51)	0.35		
rs9880772	NCI rep	g	122	3519	A/G	0.472	1.08	(0.84-1.40)	0.55		
rs9880772	MCC-Spain	g	143	140	A/G	0.439	1.34	(0.97-1.85)	0.08		
**rs9880772**	**Combined**		**5032**	**13078**	**A/G**	**0.465**	**1.19**	**(1.13-1.25)**	**2.55E-11**	**0.50**	**0**
rs9815073	NCI GWAS	i (0.842)	2178	6220	C/A	0.646	1.23	(1.15-1.35)	1.97E-07		
rs9815073	Utah GWAS	i (0.791)	321	404	C/A	0.663	1.25	(0.97-1.61)	0.08		
rs9815073	GEC GWAS	i (0.789)	386	293	C/A	0.685	1.01	(0.77-1.32)	0.97		
rs9815073	UCSF2 GWAS	i (0.790)	213	746	C/A	0.674	0.97	(0.75-1.25)	0.82		
rs9815073	Mayo/ IA-M/GEC	g	504	515	C/A	0.662	1.20	(1.00-1.45)	0.05		
rs9815073	MD Anderson	g	634	635	C/A	0.652	1.02	(0.87-1.20)	0.79		
rs9815073	MSKCC	g	285	377	C/A	0.655	1.10	(0.84-1.43)	0.49		
rs9815073	Utah-Sheffield	g	227	224	C/A	0.616	1.35	(1.01-1.79)	0.04		
rs9815073	NCI rep	g	60	2205	C/A	0.654	1.45	(0.95-2.17)	0.08		
rs9815073	MCC-Spain	g	138	138	C/A	0.645	0.98	(0.70-1.49)	0.92		
**rs9815073**	**Combined**		**4946**	**11757**	**C/A**	**0.651**	**1.18**	**(1.11-1.25)**	**3.62E-08**	**0.26**	**19.7**
rs73718779	NCI GWAS	i (0.986)	2179	6220	T/C	0.114	1.27	(1.14-1.42)	1.45E-05		
rs73718779	Utah GWAS	i (0.968)	320	404	T/C	0.099	1.54	(1.12-2.14)	0.009		
rs73718779	GEC GWAS	i (0.949)	386	293	T/C	0.109	1.20	(0.84-1.70)	0.31		
rs73718779	UCSF2 GWAS	i (0.957)	212	746	T/C	0.099	1.10	(0.76-1.59)	0.61		
rs73718779	Mayo/ IA-M/GEC	g	505	518	T/C	0.119	0.95	(0.73-1.25)	0.73		
rs73718779	MD Anderson	g	640	639	T/C	0.093	1.41	(1.09-1.83)	0.009		
rs73718779	MSKCC	g	297	378	T/C	0.114	1.21	(0.83-1.77)	0.32		
rs73718779	Utah-Sheffield	g	224	216	T/C	0.102	1.17	(0.78-1.75)	0.45		
rs73718779	NCI rep	g	63	2217	T/C	0.112	1.87	(1.18-2.96)	0.006		
rs73718779	MCC-Spain	g	142	139	T/C	0.079	0.91	(0.48-1.70)	0.76		
**rs73718779**	**Combined**		**4968**	**11770**	**T/C**	**0.110**	**1.26**	**(1.16-1.36)**	**1.97E-08**	**0.27**	**18.6**
rs10028805	NCI GWAS	g	2179	6221	G/A	0.626	1.16	(1.08-1.25)	7.65E-05		
rs10028805	Utah GWAS	g	321	405	G/A	0.628	1.15	(0.93-1.43)	0.19		
rs10028805	GEC GWAS	i (0.998)	387	293	G/A	0.638	1.12	(0.90-1.41)	0.29		
rs10028805	UCSF2 GWAS	i (0.964)	212	746	G/A	0.610	1.16	(0.93-1.45)	0.18		
rs10028805	Mayo/ IA-M/GEC	g	502	512	G/A	0.620	1.23	(1.03-1.49)	0.02		
rs10028805	MD Anderson	g	640	640	G/A	0.601	1.32	(1.12-1.54)	0.0008		
rs10028805	MSKCC	g	299	377	G/A	0.621	1.18	(0.90-1.54)	0.23		
rs10028805	Utah-Sheffield	g	229	225	G/A	0.662	0.82	(0.62-1.08)	0.16		

Supplementary Table 6. Individual study results for SNPs taken forward for replication

rs10028805	NCI rep	g	63	2213	G/A	0.622	0.84	(0.58-1.22)	0.35		
rs10028805	MCC-Spain	g	143	140	G/A	0.636	1.10	(0.65-1.28)	0.60		
**rs10028805**	**Combined**		**4975**	**11772**	**G/A**	**0.624**	**1.16**	**(1.10-1.22)**	**7.19E-08**	**0.21**	**25.0**
rs1274963	NCI GWAS	g	2179	6221	A/G	0.211	1.16	(1.08-1.27)	0.0004		
rs1274963	Utah GWAS	g	321	405	A/G	0.200	1.18	(0.91-1.54)	0.21		
rs1274963	GEC GWAS	i (0.958)	387	293	A/G	0.177	1.43	(1.10-1.89)	0.008		
rs1274963	UCSF2 GWAS	g	213	747	A/G	0.217	1.33	(1.03-1.72)	0.03		
rs1274963	Mayo/ IA-M/GEC	g	503	517	A/G	0.209	1.18	(0.69-1.05)	0.13		
rs1274963	MD Anderson	g	641	639	A/G	0.191	1.11	(0.74-1.09)	0.29		
rs1274963	MSKCC	g	299	377	A/G	0.191	1.12	(0.64-1.25)	0.50		
rs1274963	Utah-Sheffield	g	231	225	A/G	0.238	1.02	(0.71-1.33)	0.89		
rs1274963	NCI rep	g	121	3505	A/G	0.207	1.20	(0.61-1.12)	0.22		
rs1274963	MCC-Spain	g	143	139	A/G	0.147	1.05	(0.61-1.47)	0.82		
**rs1274963**	**Combined**		**5038**	**13068**	**A/G**	**0.208**	**1.18**	**(1.11-1.25)**	**2.12E-07**	**0.87**	**0**
rs6893857	NCI GWAS	g	2179	6221	C/T	0.177	1.21	(1.11-1.33)	3.02E-05		
rs6893857	Utah GWAS	g	321	405	C/T	0.201	1.18	(0.92-1.52)	0.20		
rs6893857	GEC GWAS	g	387	294	C/T	0.182	1.03	(0.77-1.36)	0.85		
rs6893857	UCSF2 GWAS	g	213	747	C/T	0.191	1.31	(1.00-1.72)	0.05		
rs6893857	Mayo/ IA-M/GEC	g	503	515	C/T	0.193	1.07	(0.86-1.34)	0.53		
rs6893857	MD Anderson	g	636	640	C/T	0.175	1.11	(0.90-1.36)	0.33		
rs6893857	Utah-Sheffield	g	225	220	C/T	0.200	1.02	(0.72-1.42)	0.93		
rs6893857	NCI rep	g	61	2191	C/T	0.193	1.26	(0.82-1.95)	0.29		
rs6893857	MCC-Spain	g	141	139	C/T	0.194	1.21	(0.55-1.25)	0.37		
**rs6893857**	**Combined**		**4666**	**11372**	**C/T**	**0.183**	**1.17**	**(1.10-1.25)**	**2.16E-06**	**0.88**	**0**
rs115819718	NCI GWAS	g	2179	6221	A/G	0.280	1.16	(1.07-1.25)	0.0002		
rs115819718	Utah GWAS	i (1.0)	321	405	A/G	0.268	1.28	(1.01-1.61)	0.04		
rs115819718	GEC GWAS	g	387	294	A/G	0.303	1.03	(0.81-1.30)	0.82		
rs115819718	UCSF2 GWAS	i (0.999)	213	747	A/G	0.257	1.25	(0.98-1.60)	0.08		
rs115819718	Mayo/ IA-M/GEC	g	504	518	A/G	0.286	1.09	(0.91-1.32)	0.35		
rs115819718	MD Anderson	g	634	639	A/G	0.290	1.11	(0.94-1.32)	0.22		
rs115819718	MSKCC	g	296	378	A/G	0.221	0.97	(0.71-1.32)	0.83		
rs115819718	Utah-Sheffield	g	231	225	A/G	0.298	1.30	(0.97-1.72)	0.08		
rs115819718	NCI rep	g	63	2210	A/G	0.282	0.85	(0.57-1.28)	0.44		
**rs115819718**	**Combined**		**4828**	**11637**	**A/G**	**0.278**	**1.14**	**(1.08-1.21)**	**4.65E-06**	**0.60**	**0**
rs76473307	NCI GWAS	i (0.994)	2179	6221	C/A	0.031	0.66	(0.53-0.83)	0.0002		
rs76473307	Utah GWAS	i (0.982)	320	405	C/A	0.033	0.39	(0.19-0.79)	0.009		
rs76473307	GEC GWAS	i (0.995)	386	293	C/A	0.053	0.50	(0.28-0.88)	0.02		
rs76473307	UCSF2 GWAS	i (0.988)	212	747	C/A	0.032	0.45	(0.23-0.89)	0.02		
rs76473307	Mayo/ IA-M/GEC	g	505	517	C/A	0.036	0.95	(0.60-1.51)	0.84		
rs76473307	MD Anderson	g	642	640	C/A	0.030	0.82	(0.51-1.32)	0.42		
rs76473307	MSKCC	g	283	377	C/A	0.028	1.66	(0.74-3.73)	0.22		
rs76473307	Utah-Sheffield	g	230	224	C/A	0.027	1.12	(0.50-2.53)	0.78		
rs76473307	NCI rep	g	120	3486	C/A	0.025	0.90	(0.39-2.08)	0.80		

Supplementary Table 6. Individual study results for SNPs taken forward for replication

**rs76473307**	**Combined**		**4877**	**12910**	**C/A**	**0.030**	**0.70**	**(0.60-0.82)**	**7.68E-06**	**0.08**	**42.9**
rs350822	NCI GWAS	i (0.919)	2178	6220	C/T	0.739	0.84	(0.77-0.91)	4.31E-05		
rs350822	Utah GWAS	i (0.931)	320	404	C/T	0.755	0.90	(0.70-1.15)	0.41		
rs350822	GEC GWAS	i (0.707)	386	293	C/T	0.753	1.12	(0.82-1.52)	0.47		
rs350822	UCSF2 GWAS	i (0.934)	212	746	C/T	0.749	0.76	(0.58-0.98)	0.04		
rs350822	Mayo/ IA-M/GEC	g	370	382	C/T	0.750	0.88	(0.91-1.44)	0.25		
rs350822	MD Anderson	g	637	631	C/T	0.746	0.84	(1.00-1.42)	0.05		
rs350822	MSKCC	g	295	373	C/T	0.724	1.07	(0.70-1.23)	0.62		
rs350822	Utah-Sheffield	g	220	225	C/T	0.729	1.14	(0.63-1.22)	0.42		
**rs350822**	**Combined**		**4618**	**9274**	**C/T**	**0.741**	**0.87**	**(0.82-0.93)**	**1.40E-05**	**0.23**	**25.1**
rs13218589	NCI GWAS	i (0.464)	2178	6220	A/G	0.037	1.72	(1.30-2.27)	0.0001		
rs13218589	Utah GWAS	i (0.405)	320	404	A/G	0.029	0.66	(0.23-1.89)	0.44		
rs13218589	GEC GWAS	i (0.435)	386	293	A/G	0.035	2.13	(0.95-4.77)	0.07		
rs13218589	UCSF2 GWAS	i (0.529)	212	746	A/G	0.032	2.78	(1.17-6.62)	0.02		
rs13218589	Mayo/ IA-M/GEC	g	376	381	A/G	0.051	1.04	(0.66-1.63)	0.87		
rs13218589	MD Anderson	g	639	641	A/G	0.051	0.98	(0.68-1.41)	0.91		
rs13218589	MSKCC	g	298	379	A/G	0.057	0.80	(0.44-1.42)	0.44		
rs13218589	Utah-Sheffield	g	230	225	A/G	0.067	0.69	(0.37-1.28)	0.24		
rs13218589	NCI rep	g	120	3485	A/G	0.048	0.94	(0.50-1.74)	0.84		
**rs13218589**	**Combined**		**4759**	**12774**	**A/G**	**0.042**	**1.22**	**(1.03-1.44)**	**0.02**	**0.009**	**60.5**
**Potential secondary signals at known loci**											
rs2953196	NCI GWAS	i (0.967)	2178	6220	G/A	0.755	1.30	(1.19-1.42)	1.71E-09		
rs2953196	Utah GWAS	i (0.942)	320	404	G/A	0.753	1.30	(1.01-1.67)	0.05		
rs2953196	GEC GWAS	i (0.953)	386	293	G/A	0.752	1.46	(1.12-1.90)	0.006		
rs2953196	UCSF2 GWAS	i (0.974)	212	746	G/A	0.757	1.21	(0.94-1.55)	0.15		
rs2953196	Mayo/ IA-M/GEC	g	503	518	G/A	0.753	1.46	(0.55-0.85)	0.0006		
rs2953196	MD Anderson	g	639	642	G/A	0.766	1.26	(0.65-0.96)	0.02		
rs2953196	MSKCC	g	297	374	G/A	0.786	1.37	(0.52-1.02)	0.06		
rs2953196	Utah-Sheffield	g	232	225	G/A	0.773	0.94	(0.79-1.44)	0.69		
rs2953196	NCI rep	g	62	2209	G/A	0.752	1.35	(0.47-1.17)	0.20		
**rs2953196**	**Combined**		**4829**	**11631**	**G/A**	**0.756**	**1.30**	**(1.22-1.38)**	**5.44E-16**	**0.56**	**0**
rs9308731	NCI GWAS	g	2179	6221	A/G	0.539	1.20	(1.12-1.29)	5.21E-07		
rs9308731	Utah GWAS	i (0.999)	321	404	A/G	0.593	0.99	(0.80-1.23)	0.95		
rs9308731	GEC GWAS	i (0.998)	387	293	A/G	0.556	1.24	(0.99-1.54)	0.06		
rs9308731	UCSF2 GWAS	i (0.998)	213	747	A/G	0.520	1.25	(1.01-1.55)	0.04		
rs9308731	Mayo/ IA-M/GEC	g	505	514	A/G	0.577	1.19	(0.70-1.00)	0.06		
rs9308731	MD Anderson	g	634	640	A/G	0.531	1.19	(0.72-0.99)	0.03		
rs9308731	MSKCC	g	296	378	A/G	0.499	1.29	(0.60-1.00)	0.05		
rs9308731	Utah-Sheffield	g	231	225	A/G	0.520	1.38	(0.55-0.96)	0.02		
rs9308731	NCI rep	g	121	3554	A/G	0.525	0.86	(0.89-1.50)	0.26		
rs9308731	MCC-Spain	g	142	137	A/G	0.609	1.80	(1.27-2.55)	0.0009		
**rs9308731**	**Combined**		**5029**	**13113**	**A/G**	**0.537**	**1.19**	**(1.13-1.26)**	**1.00E-11**	**0.07**	**43.5**

Supplementary Table 6. Individual study results for SNPs taken forward for replication

rs7578199	NCI GWAS	g	2179	6221	C/T	0.254	0.82	(0.75-0.89)	2.08E-06		
rs7578199	Utah GWAS	g	321	405	C/T	0.249	0.92	(0.72-1.17)	0.50		
rs7578199	GEC GWAS	g	387	294	C/T	0.276	0.75	(0.58-0.96)	0.02		
rs7578199	UCSF2 GWAS	g	213	747	C/T	0.252	0.91	(0.71-1.17)	0.47		
rs7578199	Mayo/ IA-M/GEC	g	506	518	C/T	0.254	0.86	(0.70-1.05)	0.14		
rs7578199	MD Anderson	g	642	642	C/T	0.249	0.79	(0.65-0.95)	0.01		
rs7578199	MSKCC	g	296	376	C/T	0.257	1.21	(0.91-1.61)	0.18		
rs7578199	Utah-Sheffield	g	231	225	C/T	0.244	0.92	(0.68-1.25)	0.61		
rs7578199	NCI rep	g	63	2219	C/T	0.258	1.03	(0.67-1.58)	0.91		
**rs7578199**	**Combined**		**4838**	**11647**	**C/T**	**0.255**	**0.85**	**(0.80-0.90)**	**7.89E-08**	**0.25**	**21.3**
rs2396718	NCI GWAS	g	2179	6221	C/T	0.092	1.28	(1.14-1.44)	3.42E-05		
rs2396718	Utah GWAS	i (0.939)	320	404	C/T	0.090	1.47	(1.04-2.09)	0.03		
rs2396718	GEC GWAS	i (0.923)	387	294	C/T	0.111	1.08	(0.76-1.54)	0.67		
rs2396718	UCSF2 GWAS	i (0.899)	212	746	C/T	0.097	1.50	(1.02-2.20)	0.04		
rs2396718	Mayo/ IA-M/GEC	g	371	380	C/T	0.097	1.15	(0.82-1.62)	0.41		
rs2396718	MD Anderson	g	637	631	C/T	0.100	1.16	(0.90-1.51)	0.25		
rs2396718	MSKCC	g	295	378	C/T	0.103	1.26	(0.86-1.86)	0.24		
**rs2396718**	**Combined**		**4401**	**9054**	**C/T**	**0.094**	**1.27**	**(1.16-1.38)**	**2.27E-07**	**0.81**	**0**

[a]Genotyped=g; Imputed=i

[b]EAF=Effect allele frequency

Supplementary Table 7. Conditional analyses for three SNPs at chromosome 2q13

SNP	Position	r^{2} *	Risk allele/ Other allele	OR[a]	P[a]	Conditional OR[b]	Conditional P[b]
rs17483466	111797458	0.03, 0.008	G/A	1.35 (1.27-1.44)	5.55E-21	1.28 (1.20-1.36)	1.00E-13
rs13401811	111616104	0.03, 0.0005	G/A	1.39 (1.30-1.49)	8.89E-21	1.33 (1.24-1.42)	2.18E-15
rs9308731	111908262	0.008, 0.0005	A/G	1.19 (1.13-1.25)	6.37E-11	1.16 (1.10-1.23)	2.03E-08

*r^2 linkage disequilibrium is based on 1000 Genomes Project and is between the SNP and the other 2 listed SNPs at the locus

[a]OR is the per allele odds ratio and *P* for the SNP from the combined unconditional meta-analysis of the discovery and replication, limited to subjects with genotypes for all three SNPs (4,770 cases, 11,544 controls).

[b]OR and *P* from the combined meta-analysis of the discovery and replication for the SNP after conditioning on the other 2 SNPs at the locus, limited to subjects with genotypes for all three SNPs (4,770 cases, 11,544 controls).

Supplementary Table 8. HaploReg results for the novel and suggestive SNPs associated with CLL

Chr	Position	r^2	D'	SNP	SiPhy cons	Promoter histone marks	Enhancer histone marks	DNAse	Protein bound	eQTL tissues	Motifs changed	Genes	DbSNP functional annotation
Query SNP: rs9880772 and variants with r^2 >= 0.8													
3	27758274	0.9	1	rs2371109							FXR,NF-Y	EOMES	3'-UTR
3	27758275	0.9	1	rs2887944							FXR,TCF4	EOMES	3'-UTR
3	27764623	1	1	rs3806624		H1, NHLF, HSMM, Huvec, GM12878, NHEK	HMEC	H1-hESC,HSMM,HUVEC,Th1,GM12891,GM19240,MCF-7,ProgFib,Urothelia,H7-hESC,HPAF,NH-A,NT2-D1	SUZ12		VDR	416bp 5' of EOMES	
3	27769551	1	1	rs34269949		H1						5.3kb 5' of EOMES	
3	27772014	1	1	rs1353286		H1, NHEK, HMEC		LNCaP,AoSMC,H9ES,PanIsletD,H7-hESC	CEBPB,SUZ12		Hic1,Smad,YY1,Znf143	7.8kb 5' of EOMES	
3	27777779	1	1	rs9880772							Gfi1,Mef2,Zfp105	14kb 5' of EOMES	
3	27779362	0.8	0.9	rs12635205							ERalpha-a,HNF4,STAT,TLX1::NFIC	15kb 5' of EOMES	
3	27783476	1	1	rs4680838				Urothelia				19kb 5' of EOMES	
3	27784997	0.9	1	rs9310852								21kb 5' of EOMES	
3	27793632	0.9	1	rs6773363							ATF3,CHD2,E2F,Egr-1,Ets,NRSF,Nrf1,Sin3Ak-20,YY1,Zfp161,Zfx,Znf143	29kb 5' of EOMES	
Query SNP: rs73718779 and variants with r^2 >= 0.8													
6	2963906	1	1	rs6939693			H1, K562				Egr-1,NF-AT1	SERPINB6	intronic
6	2964056	1	1	rs55803839			K562				HNF4,PPAR,RXRA,ZNF263	SERPINB6	intronic
6	2964361	1	1	rs12211218			K562	HMEC			HNF1	SERPINB6	intronic
6	2966404	1	1	rs150607545			K562				CDP,Evi-1,Foxq1,Ik-2,Pax-4	SERPINB6	intronic
6	2966408	0.9	1	rs199871169			K562				CDP,Evi-1,Foxq1,HNF1,Hoxd8,Ik-2,Irf,Mrg,Mxi1,NF-AT,NF-AT1,Ncx,RFX5	SERPINB6	intronic
6	2966463	1	1	rs7775025			K562				Sox	SERPINB6	intronic

Supplementary Table 8. HaploReg results for the novel and suggestive SNPs associated with CLL

Chr	Position	r^2	D'	SNP	SiPhy cons	Promoter histone marks	Enhancer histone marks	DNAse	Protein bound	eQTL tissues	Motifs changed	Genes	DbSNP functional annotation
6	2969278	1	1	rs73718779		NHLF, K562	HSMM, HepG2		ZBTB7A		Pax-6,Pou2f2	SERPINB6	intronic
6	2969632	0.9	1	rs149985242		K562	HepG2, HSMM, NHLF				Barhl1,Barx1,Barx2,Bsx,DMRT1,DMRT2,DMRT3,DMRT4,DMRT5,DMRT7,Dbx2,Dlx2,Hoxd8,Isl2,Lhx3,Msx-1,Ncx,Nkx2,Nkx6-1,Pax-6,Pou1f1,Pou2f2,Pou3f2,Pou3f3,Pou3f4,Pou6f1,Prrx2,Sox	SERPINB6	intronic
Query SNP: rs9815073 and variants with r^2 >= 0.8													
3	188115682	1	1	rs9815073			GM12878					LPP	intronic
Query SNP: rs9308731 and variants with r^2 >= 0.8													
2	111863438	0.9	1	rs7567444								AC096670.3	intronic
2	111872148	0.9	1	rs2015454				Adult_CD4_Th0			Pax-4	AC096670.3	intronic
2	111877297	0.9	1	rs4848393		GM12878, H1, Huvec		CLL,Th2			CTCF,E2F,Sin3Ak-20,UF1H3BETA,Znf143	BCL2L11	
2	111879100	1	1	rs2241845		Huvec, NHLF, K562, HepG2, HMEC, NHEK, HSMM, H1, GM12878		A549,H1-hESC,HSMM,HSMMtube,HUVEC,HepG2,K562,LNCaP,NHEK,8988T,AoSMC,GM12891,GM18507,GM19238,GM19239,H9ES,Hepatocytes,Huh-7,Ishikawa,MCF-7,Myometr,Osteobl,PanIsletD,Stellate,T-47D,iPS,Adult_CD4_Th0,Caco-2,GM06990,GM12864,HAEpiC,HBMEC,HCM,HCPEpiC,HIPEpiC,HMF,HPAEC,HRCEpiC,NHDF-neo,NHLF	PAX5C20,CCNT2		BHLHE40,Irf,NF-kappaB,NRSF,Znf143	BCL2L11	intronic
2	111879381	0.9	1	rs2241843		NHLF, K562, HepG2, HMEC, NHEK, HSMM, H1, GM12878, Huvec		GM12878,HepG2,NHEK,GM19239,GM19240,Huh-7,pHTE,SK-N-SH_RA			BHLHE40,ELF1,Hic1	BCL2L11	intronic
2	111884592	1	1	rs59854799							Cdx,Elf3,Evi-1,Foxp1,GATA,HDAC2,Hoxa9,NF-AT,TCF4	BCL2L11	intronic
2	111886914	1	1	rs6758181							Nkx2,Nkx3	BCL2L11	intronic

Supplementary Table 8. HaploReg results for the novel and suggestive SNPs associated with CLL

Chr	Position	r^2	D'	SNP	SiPhy cons	Promoter histone marks	Enhancer histone marks	DNAse	Protein bound	eQTL tissues	Motifs changed	Genes	DbSNP functional annotation
2	111892984	1	1	rs6746608			HepG2	GM12878,HepG2,Medullo,Urothelia,HL-60,HMVEC-LLy,HMVEC-dAd,HMVEC-dLy-Ad,HMVEC-dNeo	ELF1		BCL,Ets,RREB-1,UF1H3BETA,Zfp281	BCL2L11	intronic
2	111893869	1	1	rs6750599							NERF1a,SIX5,SZF1-1,p300	BCL2L11	intronic
2	111900598	1	1	rs13396983							CIZ,HDAC2	BCL2L11	intronic
2	111904541	1	1	rs1980045								BCL2L11	intronic
2	111906510	1	1	rs1877331			GM12878, K562, Huvec	HCPEpiC			ELF1,Ehf,Elf3,Ets,GR,Irf,Mef2,PU.1,RXRA,Zfp105,p300	BCL2L11	intronic
2	111907214	1	1	rs6542334		K562	Huvec				Fox,Foxa,Foxc1,Foxj2,Foxk1,HDAC2,Pou2f2,p300	BCL2L11	intronic
2	111908262	1	1	rs9308731			Huvec				CDP,HNF6,Pbx-1	BCL2L11	intronic
2	111912681	0.9	1	rs616130			HMEC		HNF4A			BCL2L11	intronic
2	111912715	0.8	1	rs616084			HMEC				Hltf	BCL2L11	intronic
2	111912718	0.8	1	rs676119			HMEC				Elf5	BCL2L11	intronic
2	111913737	1	1	rs3838220			HMEC, GM12878, NHEK				Foxp1,GR,LUN-1,RP58	BCL2L11	intronic
2	111913998	1	1	rs59403143			HMEC, GM12878, NHEK				Hbp1,Ik-2,Mef2,VDR	BCL2L11	intronic
2	111920741	1	1	rs3827536			NHLF, Huvec, HSMM, GM12878	HSMMtube,GM12892,GM19238,PanIslets,Stellate,Urothelia,CD34+_Mobilized,Caco-2,HBMEC,HL-60,HRCEpiC,HRE,Monocytes-CD14+_RO01746,SAEC,SK-N-SH_RA	CFOS,GATA2,POL2B,FOXA2,CCNT2,GABP,TAL1,P300		AP-1,BDP1,Nrf-2	BCL2L11	intronic
2	111923630	0.9	1	rs6753785				Fibrobl			GR	BCL2L11	3'-UTR
2	111928373	0.9	1	rs36018702							GATA	3.8kb 3' of BCL2L11	
2	111934219	0.9	1	rs4849442		HepG2	HMEC	A549,HMEC,HeLa-S3,HepG2,Gliobla,PanIslets,pHTE,Adult_CD4_Th0,Caco-2	GR,FOSL2,JUND,RXRA		Foxp1,Myb	9.6kb 3' of BCL2L11	
Query SNP: rs10028805 and variants with r^2 >= 0.8													
4	102736456	1	1	rs13136297							AP-2,Pbx3,SMC3	BANK1	intronic
4	102737250	1	1	rs10028805							BATF,HNF4,Hoxb13,Irf,TATA	BANK1	intronic

Supplementary Table 8. HaploReg results for the novel and suggestive SNPs associated with CLL

Chr	Position	r^2	D'	SNP	SiPhy cons	Promoter histone marks	Enhancer histone marks	DNAse	Protein bound	eQTL tissues	Motifs changed	Genes	DbSNP functional annotation
4	102737936	1	1	rs4615176							Dbx1,Nrf-2,Sox	BANK1	intronic
4	102738147	1	1	rs4411998							Arid3a,Hoxd10,Lhx3	BANK1	intronic
4	102743687	1	1	rs13136219							Homez,PLZF,Pou2f2	BANK1	intronic
4	102743811	0.9	1	rs13112246							EBF,Pax-5	BANK1	intronic
4	102744092	0.9	1	rs13137133								BANK1	intronic
4	102746780	1	1	rs4276281							BCL,ELF1,Ehf,Elf5,Ets,FEV,GATA,PU.1,Tel2	BANK1	intronic
4	102747265	1	1	rs7698632							Nkx2	BANK1	intronic
4	102747927	0.9	1	rs12163856							Foxj2,Pou2f2,Pou3f3	BANK1	intronic
4	102751276	0.9	1	rs10516486							Dbx1,HNF1,Hlx1,Hoxd8,Ncx,Nkx6-1,Pou1f1,Pou3f2,Pou4f3,STAT,Sox	BANK1	synonymous
4	102755378	0.9	1	rs13145909					GATA3,P300			BANK1	intronic
4	102756099	1	1	rs4643809							Pou3f2,TATA	BANK1	intronic
4	102757065	0.9	1	rs7682827							NF-I,Pou2f2,Sox,YY1,p300	BANK1	intronic
4	102757578	0.9	1	rs10016325							ATF3,HEN1,Rad21,Zbtb3	BANK1	intronic
4	102762581	1	1	rs10032160							Foxp1,Pou2f2,Pou3f2,Pou3f3	BANK1	intronic
Query SNP: rs1274963 and variants with r^2 >= 0.8													
3	39190775	1	1	rs1274964			GM12878, Huvec, K562, NHEK, HSMM, HMEC, NHLF	GM12878,GM19238,GM19240,CD20+,CD34+_Mobilized,GM12865,Monocytes-CD14+_RO01746	EBF1,OCT2,POL2,POU2F2,NFKB		EBF,GR,MAZ,RXRA,Rad21,SMC3,SP1,Zic	CSRNP1	
3	39191029	1	1	rs1274963			GM12878, Huvec, K562, NHLF, NHEK, HSMM, HMEC	K562,Hepatocytes	POL24H8		BDP1,EWSR1-FLI1,Maf,Myf,TCF12	CSRNP1	
3	39191335	1	1	rs1274961			GM12878, Huvec, K562, HSMM, HMEC, NHEK, NHLF	HMVEC-dNeo	POL24H8		GR,Hsf	CSRNP1	intronic

Supplementary Table 9. Meta-analysis eQTL results for whole blood for the CLL-associated SNPs and SNPs in LD (r^2>.8)*[*]

Locus	CLL-associated SNP	SNP in LD	r^2	SNP location	Probe location	Gene name	Minor/Major allele	Z-score	P-value
New loci									
6p25.2	rs73718779	rs6939693	1	6:2908905	6:2893438	*SERPINB6*	T/C	-15.26	1.40E-52
6p25.2	rs73718779	rs6939693	1	6:2908905	6:2935777	*AL133351.34-1*	T/C	-5.65	1.61E-08
New suggestive loci									
3p22.2	rs1274963	rs1274963	1	3:39166033	3:39112675	*WDR48*	A/G	-16.98	1.13E-64
3p22.2	rs1274963	rs1274963	1	3:39166033	3:39113436	*GORASP1*	A/G	-4.61	4.01E-06

[*]Only eQTL associations with FDR < 0.01 are shown.

Supplementary Table 10. MeQTL association results for new and suggestive CLL loci*

Locus	CLL SNP	SNP in LD	r^2	CpG	CpG position	Distance from SNP to CpG site	Genes	CpG island/ Shore status	Effect allele/ Other allele	Beta	P
**New loci**											
6p25.2	rs73718779	rs6939693	1	cg15861059	2972195	-8289	*SERPINB6*	S_Shore	C/T	-0.0174	1.70E-11
6p25.2	rs73718779	rs6939693	1	cg06945625	2972184	-8278	*SERPINB6*	S_Shore	C/T	-0.0179	1.47E-10
6p25.2	rs73718779	rs6939693	1	cg21995203	2972180	-8274	*SERPINB6*	S_Shore	C/T	-0.0120	1.89E-10
6p25.2	rs73718779	rs6939693	1	cg09841323	2972158	-8252	*SERPINB6*	S_Shore	C/T	-0.0096	5.25E-09
6p25.2	rs73718779	rs6939693	1	cg14248680	2972186	-8280	*SERPINB6*	S_Shore	C/T	-0.0124	5.85E-07
6p25.2	rs73718779	rs6939693	1	cg06183820	2972097	-8191	*SERPINB6*	S_Shore	C/T	-0.0013	0.0007
**New independent SNP in known locus**											
2q13	rs9308731	rs7567444	1	cg11842141	111878481	-15043	*BCL2L11*	Island	C/T	0.0006	0.006
2q13	rs9308731	rs2015454	1	cg11842141	111878481	-6333	*BCL2L11*	Island	G/A	0.0006	0.005
2q13	rs9308731	rs6746608	1	cg11842141	111878481	14503	*BCL2L11*	Island	G/A	0.0006	0.006
2q13	rs9308731	rs13396983	1	cg11842141	111878481	22117	*BCL2L11*	Island	G/A	0.0006	0.007
2q13	rs9308731	rs1980045	1	cg11842141	111878481	26060	*BCL2L11*	Island	G/A	0.0006	0.007
2q13	rs9308731	rs1877331	1	cg11842141	111878481	28029	*BCL2L11*	Island	A/G	0.0006	0.007
2q13	rs9308731	rs9308731	1	cg11842141	111878481	29781	*BCL2L11*	Island	A/G	0.0006	0.007
2q13	rs9308731	rs616130	1	cg11842141	111878481	34200	*BCL2L11*	Island	A/C	0.0006	0.006
2q13	rs9308731	rs6753785	1	cg11842141	111878481	45149	*BCL2L11*	Island	G/T	0.0005	0.009
**New suggestive loci**											
3p22.2	rs1274963	rs1274963	1	cg14694744	39234137	-43108	*XIRP1*		A/G	-0.0102	4.90E-06

*Results are restricted to CpG sites within 200bp of the transcription start site of a gene.

Supplementary Table 11. Significant eQTL association results for new and suggestive loci based on lymphoblastoid cells from childhood asthma study*

Locus	CLL SNP	Gene transcript	Effect allele	Other allele	Beta for CLL SNP[a]	P for CLL SNP[a]	$P_{conditioned\ on\ peak\ SNP}$[b]	Peak SNP for transcript[c]	r^2	Beta for Peak SNP[d]	P for Peak SNP[d]	$P_{conditioned\ on\ CLL\ SNP}$[e]
New independent SNP at known locus												
2q13	rs9308731	*METAP1D*	A	G	0.2083	4.30E-05	1.31E-05	rs146548287	-	-14.1144	9.21E-07	3.59E-07
New suggestive loci												
4q24	rs10028805	*BANK1*	G	A	0.3472	6.89E-13	0.002	rs7686702	0.698	-0.3554	7.61E-14	0.0003
3p22.2	rs1274963	*WDR48*	G	A	0.1816	2.51E-05	0.02	rs2056613	0.102	-0.4172	2.26E-30	7.81E-29

*Only *cis* associations that reached $P<6.8\times10^{-5}$, which corresponds to a false-discovery rate (FDR) of 1% are reported.

[a]Beta and p-value for the association between the CLL SNP and gene transcript.

[b]p-value for the association between the CLL SNP and gene transcript after adjustment for the peak SNP

[c]Peak SNP is the most significant SNP associated with the gene transcript

[d]Beta and p-value for the association between the peak SNP and the gene transcript

[e]P-value for the association between the peak SNP and the gene transcript after adjustment for the CLL SNP

Supplementary Table 12. Results from GRAIL from new and previously reported loci

Region	SNP	GRAIL p-value	Candidate gene(s)
Region26	rs4368253	3.23E-08	*PMAIP1*
Region2	rs17483466	1.31E-07	*BCL2L11*
Region27	rs4987855	2.08E-07	*BCL2*
Region13	rs210142	2.44E-06	*BAK1*
Region22	rs8024033	2.73E-06	*BMF*
Region5	rs757978	1.39E-05	*BOK*
Region9	rs10069690	1.47E-05	*TERT*
Region25	rs305061	9.64E-05	*IRF8*
Region19	rs4406737	0.004	*FAS*
Region11	rs872071	0.004	*IRF4*
Region3	rs3769825	0.005	*CASP8*
Region7	rs10936599	0.007	*TERC*
Region15	rs17246404	0.02	*POT1*
Region10	rs73718779	0.05	*SERPINB9*
Region6	rs9880772	0.12	*EOMES*
Region12	rs9273363	0.17	*HLA-DQB2*
Region1	rs3770745	0.19	*EIF2AK2*
Region20	rs7944004	0.47	*TH*
Region8	rs898518	0.48	*LEF1*
Region4	rs13397985	0.50	*SP110*
Region28	rs11083846	0.59	*PTGIR*
Region16	rs2511714	0.68	*KLF10*
Region29	rs9815073	0.69	*LPP*
Region21	rs735665	0.71	*ZNF202*
Region17	rs2456449	0.85	*POU5F1P1*
Region30	rs10735079	0.86	*DTX1*
Region14	rs2236256	0.91	*CNKSR3*
Region18	rs1679013	0.92	*CDKN2B*
Region23	rs11636802	0.93	*MNS1*
Region24	rs7176508	N/A	

Keywords Describing Functional Connections	
'apoptosis'	'cell'
'telomerase'	'proapoptotic'
'death'	'telomeric'
'caspase'	'activation'
'apoptotic'	'hydroxylase'
'telomere'	'stranded'
'interferon'	'mediated'
'cells'	'response'
'induced'	'lymphoid'
'mice'	'infection'

Supplementary Table 13. Most significant GeneMania results for new and known CLL loci*

Pathway or biological feature	FDR	No. of genes in network	No. of genes in genome
regulation of apoptotic signaling pathway	2.06E-17	17	201
regulation of mitochondrial membrane permeability	2.35E-17	12	49
mitochondrial outer membrane	7.18E-17	13	78
regulation of mitochondrial outer membrane permeabilization involved in apoptotic signaling pathway	7.52E-17	11	38
outer membrane	1.72E-16	13	90
mitochondrial membrane organization	1.72E-16	12	64
positive regulation of mitochondrial membrane permeability	1.72E-16	11	44
mitochondrial outer membrane permeabilization	1.72E-16	11	43
positive regulation of mitochondrial membrane permeability involved in apoptotic process	1.72E-16	11	44
organelle outer membrane	1.72E-16	13	88
mitochondrial outer membrane permeabilization involved in programmed cell death	1.72E-16	11	44
signal transduction in absence of ligand	2.34E-16	11	46
regulation of mitochondrial membrane permeability involved in apoptotic process	2.34E-16	11	46
extrinsic apoptotic signaling pathway in absence of ligand	2.34E-16	11	46
extrinsic apoptotic signaling pathway	4.93E-16	14	136
intrinsic apoptotic signaling pathway	5.50E-16	15	180
positive regulation of mitochondrion organization	1.09E-15	11	53
regulation of mitochondrion organization	1.37E-15	12	79
apoptotic mitochondrial changes	2.43E-15	12	83
regulation of protein insertion into mitochondrial membrane involved in apoptotic signaling pathway	7.82E-15	9	26
positive regulation of protein insertion into mitochondrial membrane involved in apoptotic signaling pathway	7.82E-15	9	26
protein insertion into mitochondrial membrane involved in apoptotic signaling pathway	7.82E-15	9	26
protein insertion into mitochondrial membrane	1.12E-14	9	27
protein insertion into membrane	1.19E-13	9	34
release of cytochrome c from mitochondria	7.50E-13	9	41
positive regulation of intrinsic apoptotic signaling pathway	2.94E-12	8	28
regulation of intrinsic apoptotic signaling pathway	5.06E-12	10	78
positive regulation of apoptotic signaling pathway	1.67E-11	9	57
positive regulation of organelle organization	2.02E-10	12	216
establishment of protein localization to mitochondrion	2.37E-10	9	76
mitochondrion organization	3.07E-10	12	225
mitochondrial membrane	3.09E-10	13	294
regulation of release of cytochrome c from mitochondria	1.09E-09	7	32
establishment of protein localization to membrane	1.12E-09	12	253
regulation of extrinsic apoptotic signaling pathway	1.20E-09	9	92
regulation of extrinsic apoptotic signaling pathway in absence of ligand	1.59E-09	7	34
positive regulation of cysteine-type endopeptidase activity	2.45E-09	9	100
positive regulation of endopeptidase activity	3.33E-09	9	104
regulation of mitochondrial membrane potential	3.33E-09	6	19
positive regulation of peptidase activity	4.47E-09	9	108
positive regulation of release of cytochrome c from mitochondria	4.47E-09	6	20

Supplementary Table 13. Most significant GeneMania results for new and known CLL loci*

Pathway or biological feature	FDR	No. of genes in network	No. of genes in genome
regulation of cysteine-type endopeptidase activity	4.71E-09	10	159
intrinsic apoptotic signaling pathway in response to DNA damage	7.03E-09	8	73
activation of cysteine-type endopeptidase activity	1.46E-08	8	80
regulation of endopeptidase activity	1.62E-08	11	248
regulation of peptidase activity	2.14E-08	11	255
positive regulation of cysteine-type endopeptidase activity involved in apoptotic process	4.29E-08	8	92
regulation of intracellular transport	4.98E-08	11	277
zymogen activation	5.81E-08	8	96
regulation of cysteine-type endopeptidase activity involved in apoptotic process	5.98E-08	9	147
regulation of establishment of protein localization	9.84E-08	11	297
positive regulation of protein oligomerization	1.15E-07	5	15
activation of cysteine-type endopeptidase activity involved in apoptotic process	3.34E-07	7	74
regulation of protein oligomerization	1.22E-06	5	23
cellular response to external stimulus	2.19E-05	7	135
cellular response to mechanical stimulus	2.52E-05	5	41
regulation of execution phase of apoptosis	3.39E-05	4	17
cellular response to abiotic stimulus	5.84E-05	7	157
negative regulation of signal transduction in absence of ligand	6.51E-05	4	20
negative regulation of extrinsic apoptotic signaling pathway in absence of ligand	6.51E-05	4	20
positive regulation of apoptotic process	8.76E-05	8	250
anoikis	9.48E-05	4	22
positive regulation of programmed cell death	9.87E-05	8	255
positive regulation of cell death	1.46E-04	8	269
protein heterodimerization activity	1.90E-04	7	190
response to mechanical stimulus	2.43E-04	5	66
endoplasmic reticulum calcium ion homeostasis	5.92E-04	3	10
regulation of protein complex assembly	8.21E-04	6	152
positive regulation of protein complex assembly	0.001	5	89
regulation of protein homodimerization activity	0.001	3	12
extrinsic apoptotic signaling pathway via death domain receptors	0.001	4	41
regulation of membrane potential	0.001	6	165
regulation of protein homooligomerization	0.001	3	13
negative regulation of apoptotic signaling pathway	0.001	5	94
execution phase of apoptosis	0.002	5	99
pore complex	0.002	4	47
negative regulation of anoikis	0.002	3	15
negative regulation of intrinsic apoptotic signaling pathway	0.002	4	49
protease binding	0.002	4	49
mitochondrial transport	0.002	5	107
negative regulation of extrinsic apoptotic signaling pathway	0.003	4	53
cell-type specific apoptotic process	0.003	6	194
response to endoplasmic reticulum stress	0.003	5	113

Supplementary Table 13. Most significant GeneMania results for new and known CLL loci*

Pathway or biological feature	FDR	No. of genes in network	No. of genes in genome
embryonic placenta development	0.003	3	18
regulation of anoikis	0.004	3	19
positive regulation of proteolysis	0.006	4	65
leukocyte differentiation	0.006	6	226
hemopoiesis	0.007	6	232
negative regulation of intracellular signal transduction	0.007	6	234
alpha-beta T cell differentiation involved in immune response	0.008	3	25
alpha-beta T cell activation involved in immune response	0.008	3	25
regulation of protein binding	0.008	4	72
T cell activation	0.008	6	241
interferon-gamma-mediated signaling pathway	0.008	4	73
positive regulation of protein processing	0.008	4	73
cellular response to type I interferon	0.009	4	74
type I interferon signaling pathway	0.009	4	74
response to type I interferon	0.009	4	75
T cell differentiation involved in immune response	0.009	3	27
hematopoietic or lymphoid organ development	0.009	6	250
regulation of proteolysis	0.01	5	155
sequence-specific DNA binding	0.01	6	254
positive regulation of myeloid leukocyte differentiation	0.01	3	28
positive regulation of extrinsic apoptotic signaling pathway	0.01	3	29
immune system development	0.01	6	266
cellular response to interferon-gamma	0.02	4	89
T cell differentiation	0.02	4	90
placenta development	0.02	3	35
alpha-beta T cell differentiation	0.02	3	35
myeloid leukocyte differentiation	0.02	4	94
adaptive immune response based on somatic recombination of immune receptors built from immunoglobulin superfamily domains	0.02	4	98
defense response to other organism	0.02	5	188
protein oligomerization	0.03	5	194
response to interferon-gamma	0.03	4	105
regulation of protein processing	0.03	5	198
regulation of leukocyte differentiation	0.03	4	109
response to virus	0.03	5	205
antigen receptor-mediated signaling pathway	0.04	4	114
positive regulation of myeloid cell differentiation	0.04	3	46
defense response to virus	0.04	4	116
protein homooligomerization	0.04	4	116
in utero embryonic development	0.04	3	47
nucleotide-binding domain, leucine rich repeat containing receptor signaling pathway	0.04	3	47
alpha-beta T cell activation	0.05	3	49
response to nicotine	0.05	2	10

Supplementary Table 13. Most significant GeneMania results for new and known CLL loci[*]

Pathway or biological feature	FDR	No. of genes in network	No. of genes in genome
T cell homeostasis	0.05	2	10
regulation of interleukin-13 production	0.05	2	10
T cell activation involved in immune response	0.05	3	51

[*]Created on: 23 December 2014 with GeneMania Application version: 3.1.2.8

Supplementary Table 14. WEB-based Gene SeT Analysis results for new and known CLL loci

Translating gene lists into biological insights...

The results for the enriched GO category are listed in this table. For each GO category, the first row lists its sub-root (biological process, molecular function, or cellular component), category name, and corresponding GO ID. The second row lists the following statistics:

C: the number of reference genes in the category
O: the number of genes in the gene set and also in the category
E: the expected number in the category
R: ratio of enrichment
rawP: p value from hypergeometric test
adjP: p value adjusted by the multiple test adjustment

Finally, genes in the category are listed. For each gene, the table lists the user uploaded ID and value (optional), Entrez ID, Ensembl Gene Stable ID, Gene symbol, and description. Ensembl Gene Stable ID and Entrez Gene ID are linked to the Ensembl and Entrez Gene databases, respectively.

**Database:biological process**	**Name:activation of pro-apoptotic gene products**				**ID:GO:0008633**	
C=31; O=7; E=0.06; R=122.35; rawP=7.98e-14; adjP=5.49e-11						
Index	UserID	Value	Gene Symbol	Gene Name	EntrezGene	Ensembl
1	BAK1	NA	BAK1	BCL2- antagonist/killer 1	578	ENSG00000030110
2	BCL2	NA	BCL2	B-cell CLL/lymphoma 2	596	ENSG00000171791
3	BMF	NA	BMF	Bcl2 modifying factor	90427	ENSG00000104081
4	BCL2L11	NA	BCL2L11	BCL2-like 11 (apoptosis facilitator)	10018	ENSG00000153094
5	CASP8	NA	CASP8	caspase 8, apoptosis- related cysteine peptidase	841	ENSG00000064012
6	PMAIP1	NA	PMAIP1	phorbol-12- myristate-13- acetate-induced protein 1	5366	ENSG00000141682
7	FAS	NA	FAS	Fas (TNF receptor superfamily, member 6)	355	ENSG00000026103

**Database:biological process**	**Name:release of cytochrome c from mitochondria**				**ID:GO:0001836**	
C=46; O=5; E=0.08; R=58.90; rawP=1.88e-08; adjP=2.16e-06						
Index	UserID	Value	Gene Symbol	Gene Name	EntrezGene	Ensembl
1	BAK1	NA	BAK1	BCL2- antagonist/killer 1	578	ENSG00000030110
2	BCL2	NA	BCL2	B-cell CLL/lymphoma 2	596	ENSG00000171791
3	BMF	NA	BMF	Bcl2 modifying factor	90427	ENSG00000104081
4	BCL2L11	NA	BCL2L11	BCL2-like 11 (apoptosis facilitator)	10018	ENSG00000153094
5	PMAIP1	NA	PMAIP1	phorbol-12- myristate-13- acetate-induced protein 1	5366	ENSG00000141682

**Database:biological process**	**Name:apoptotic signaling pathway**				**ID:GO:0097190**	
C=169; O=7; E=0.31; R=22.44; rawP=1.77e-08; adjP=2.16e-06						
Index	UserID	Value	Gene Symbol	Gene Name	EntrezGene	Ensembl
1	BAK1	NA	BAK1	BCL2- antagonist/killer 1	578	ENSG00000030110
2	BCL2	NA	BCL2	B-cell CLL/lymphoma 2	596	ENSG00000171791
3	BMF	NA	BMF	Bcl2 modifying factor	90427	ENSG00000104081
4	BCL2L11	NA	BCL2L11	BCL2-like 11 (apoptosis facilitator)	10018	ENSG00000153094
5	CASP8	NA	CASP8	caspase 8, apoptosis- related cysteine peptidase	841	ENSG00000064012
6	PMAIP1	NA	PMAIP1	phorbol-12- myristate-13- acetate-induced protein 1	5366	ENSG00000141682
7	FAS	NA	FAS	Fas (TNF receptor superfamily, member 6)	355	ENSG00000026103

**Database:biological process**	**Name:lymphocyte homeostasis**	**ID:GO:0002260**				
C=44; O=5; E=0.08; R=61.57; rawP=1.50e-08; adjP=2.16e-06						
Index	UserID	Value	Gene Symbol	Gene Name	EntrezGene	Ensembl
1	BAK1	NA	BAK1	BCL2- antagonist/killer 1	578	ENSG00000030110
2	BCL2	NA	BCL2	B-cell CLL/lymphoma 2	596	ENSG00000171791
3	BCL2L11	NA	BCL2L11	BCL2-like 11 (apoptosis facilitator)	10018	ENSG00000153094
4	PMAIP1	NA	PMAIP1	phorbol-12- myristate-13- acetate-induced protein 1	5366	ENSG00000141682
5	FAS	NA	FAS	Fas (TNF receptor superfamily, member 6)	355	ENSG00000026103

**Database:biological process**	**Name:regulation of execution phase of apoptosis**	**ID:GO:1900117**				
C=169; O=7; E=0.31; R=22.44; rawP=1.77e-08; adjP=2.16e-06						
Index	UserID	Value	Gene Symbol	Gene Name	EntrezGene	Ensembl
1	BAK1	NA	BAK1	BCL2-antagonist/killer 1	578	ENSG00000030110
2	BCL2	NA	BCL2	B-cell CLL/lymphoma 2	596	ENSG00000171791
3	BMF	NA	BMF	Bcl2 modifying factor	90427	ENSG00000104081
4	BCL2L11	NA	BCL2L11	BCL2-like 11 (apoptosis facilitator)	10018	ENSG00000153094
5	CASP8	NA	CASP8	caspase 8, apoptosis- related cysteine peptidase	841	ENSG00000064012
6	PMAIP1	NA	PMAIP1	phorbol-12- myristate-13- acetate-induced protein 1	5366	ENSG00000141682
7	FAS	NA	FAS	Fas (TNF receptor superfamily, member 6)	355	ENSG00000026103

**Database:biological process**	**Name:positive regulation of protein oligomerization**	**ID:GO:0032461**				
C=16; O=4; E=0.03; R=135.46; rawP=1.65e-08; adjP=2.16e-06						
Index	UserID	Value	Gene Symbol	Gene Name	EntrezGene	Ensembl
1	BMF	NA	BMF	Bcl2 modifying factor	90427	ENSG00000104081
2	BCL2L11	NA	BCL2L11	BCL2-like 11 (apoptosis facilitator)	10018	ENSG00000153094
3	PMAIP1	NA	PMAIP1	phorbol-12- myristate- 13-acetate- induced protein 1	5366	ENSG00000141682
4	FAS	NA	FAS	Fas (TNF receptor superfamily, member 6)	355	ENSG00000026103

**Database:biological process**	**Name:positive regulation of release of cytochrome c from mitochondria**	**ID:GO:0090200**				
C=20; O=4; E=0.04; R=108.37; rawP=4.37e-08; adjP=3.76e-06						
Index	UserID	Value	Gene Symbol	Gene Name	EntrezGene	Ensembl
1	BAK1	NA	BAK1	BCL2- antagonist/killer 1	578	ENSG00000030110
2	BMF	NA	BMF	Bcl2 modifying factor	90427	ENSG00000104081
3	BCL2L11	NA	BCL2L11	BCL2-like 11 (apoptosis facilitator)	10018	ENSG00000153094
4	PMAIP1	NA	PMAIP1	phorbol-12- myristate-13- acetate-induced protein 1	5366	ENSG00000141682

**Database:biological process**	**Name:leukocyte homeostasis**	**ID:GO:0001776**				
C=53; O=5; E=0.10; R=51.12; rawP=3.91e-08; adjP=3.76e-06						
Index	UserID	Value	Gene Symbol	Gene Name	EntrezGene	Ensembl
1	BAK1	NA	BAK1	BCL2- antagonist/killer 1	578	ENSG00000030110
2	BCL2	NA	BCL2	B-cell CLL/lymphoma 2	596	ENSG00000171791
3	BCL2L11	NA	BCL2L11	BCL2-like 11 (apoptosis facilitator)	10018	ENSG00000153094
4	PMAIP1	NA	PMAIP1	phorbol-12- myristate-13- acetate-induced	5366	ENSG00000141682

				protein 1		
5	FAS	NA	FAS	Fas (TNF receptor superfamily, member 6)	355	ENSG00000026103

Database:biological process			Name:apoptotic mitochondrial changes		ID:GO:0008637	
C=58; O=5; E=0.11; R=46.71; rawP=6.20e-08; adjP=4.74e-06						
Index	UserID	Value	Gene Symbol	Gene Name	EntrezGene	Ensembl
1	BAK1	NA	BAK1	BCL2- antagonist/killer 1	578	ENSG00000030110
2	BCL2	NA	BCL2	B-cell CLL/lymphoma 2	596	ENSG00000171791
3	BMF	NA	BMF	Bcl2 modifying factor	90427	ENSG00000104081
4	BCL2L11	NA	BCL2L11	BCL2-like 11 (apoptosis facilitator)	10018	ENSG00000153094
5	PMAIP1	NA	PMAIP1	phorbol-12- myristate-13- acetate-induced protein 1	5366	ENSG00000141682

Database:biological process			Name:regulation of protein oligomerization		ID:GO:0032459	
C=23; O=4; E=0.04; R=94.24; rawP=7.95e-08; adjP=4.97e-06						
Index	UserID	Value	Gene Symbol	Gene Name	EntrezGene	Ensembl
1	BMF	NA	BMF	Bcl2 modifying factor	90427	ENSG00000104081
2	BCL2L11	NA	BCL2L11	BCL2-like 11 (apoptosis facilitator)	10018	ENSG00000153094
3	PMAIP1	NA	PMAIP1	phorbol-12- myristate- 13-acetate- induced protein 1	5366	ENSG00000141682
4	FAS	NA	FAS	Fas (TNF receptor superfamily, member 6)	355	ENSG00000026103

Database:cellular componenet			Name:mitochondrial outer membrane		ID:GO:0005741	
C=122; O=6; E=0.21; R=28.92; rawP=4.92e-08; adjP=3.89e-06						
Index	UserID	Value	Gene Symbol	Gene Name	EntrezGene	Ensembl
1	BAK1	NA	BAK1	BCL2-antagonist/killer 1	578	ENSG00000030110
2	BCL2	NA	BCL2	B-cell CLL/lymphoma 2	596	ENSG00000171791
3	BMF	NA	BMF	BCL3 modifying factor	90427	ENSG00000104081
4	BCL2L11	NA	BCL2L11	BCL-like11	10018	ENSG00000153094
5	CASP8	NA	CASP8	caspase 8, apoptosis-	841	ENSG00000064012
6	PMAIP	NA	PMAIP1	phorbol-12- myristate-13- acetate-induced protein 1	5366	ENSG00000141682

Database:cellular component			Name:organelle outer membrane		ID:GO:0031968	
C=143; O=6; E=0.24; R=24.67; rawP=1.27e-07; adjP=4.27e-06						
Index	UserID	Value	Gene Symbol	Gene Name	EntrezGene	Ensembl
1	BAK1	NA	BAK1	BCL2- antagonist/killer 1	578	ENSG00000030110
2	BCL2	NA	BCL2	B-cell CLL/lymphoma 2	596	ENSG00000171791
3	BMF	NA	BMF	Bcl2 modifying factor	90427	ENSG00000104081
4	BCL2L11	NA	BCL2L11	BCL2-like 11 (apoptosis facilitator)	10018	ENSG00000153094
5	CASP8	NA	CASP8	caspase 8, apoptosis- related cysteine peptidase	841	ENSG00000064012
6	PMAIP1	NA	PMAIP1	phorbol-12- myristate-13- acetate-induced protein 1	5366	ENSG00000141682

Database:cellular component			Name:outer membrane		ID:GO:0019867	
C=149; O=6; E=0.25; R=23.68; rawP=1.62e-07; adjP=4.27e-06						
Index	UserID	Value	Gene Symbol	Gene Name	EntrezGene	Ensembl
1	BAK1	NA	BAK1	BCL2- antagonist/killer 1	578	ENSG00000030110

2	BCL2	NA	BCL2	B-cell CLL/lymphoma 2	596	ENSG00000171791
3	BMF	NA	BMF	Bcl2 modifying factor	90427	ENSG00000104081
4	BCL2L11	NA	BCL2L11	BCL2-like 11 (apoptosis facilitator)	10018	ENSG00000153094
5	CASP8	NA	CASP8	caspase 8, apoptosis- related cysteine peptidase	841	ENSG00000064012
6	PMAIP1	NA	PMAIP1	phorbol-12- myristate-13- acetate-induced protein 1	5366	ENSG00000141682

WebGestalt is currently developed and maintained by Jing Wang and Bing Zhang at the Zhang Lab. Other people who have made significant contribution to the project include Dexter Duncan, Stefan Kirov, Zhiao Shi, and Jay Snoddy.
Funding credits: NIH/NIAAA (U01 AA016662, U01 AA013512); NIH/NIDA (P01 DA015027); NIH/NIMH (P50 MH078028, P50 MH096972); NIH/NCI (U24 CA159988); NIH/NIGMS (R01 GM088822).
